# Physico-chemical factors and nanomaterial-mediated optimization of pigment yield from a psychrotrophic marine *Citricoccus-*like isolate of an Arctic fjord

**DOI:** 10.3389/fbioe.2026.1824301

**Published:** 2026-09-25

**Authors:** Rakshita Kaskar, Manisha Tiwari, Meghanath Prabhu, Savita Kerkar, Dhermendra K. Tiwari

**Affiliations:** 1 School of Biological Sciences and Biotechnology, Goa University, Taleigao, Goa, India; 2 University Grant Commission (UGC), New Delhi, India

**Keywords:** Arctic fjord, bacterial pigment, *Citricoccus* sp., nanomaterial, response surface methodology, TiO_2_

## Abstract

Bacterial pigments have broad industrial applications and are relatively easy to scale up. However, Arctic microbial sources remain largely unexplored. In this study, we isolated a yellow-pigmented bacterium, PS104, from the Arctic Ny-Ålesund fjord in Kongsfjorden, Svalbard, Norway. Biochemical assays and 16S rRNA gene sequencing revealed that PS104 is closely related to the genus *Citricoccus*. Pigment characterization using UV–visible spectroscopy showed a carotenoid-like profile with a three-shoulder absorption peak, while Fourier transform infrared (FT-IR) spectroscopy and high-resolution liquid chromatography mass spectrophotometry (HR-LCMS) analysis confirmed the presence of yellow carotenoid pigment consistent with zeaxanthin (C_40_H_56_O_2_), along with additional unidentified peaks. To enhance the pigment yield, we employed a multi-stage optimization strategy beginning with one-factor-at-a-time, followed by response surface methodology (RSM), which resulted in ∼200% increases in pigment dry weight from 26 mg/100 mL to 78 mg/100 mL. Further supplementation of RSM media with TiO_2_ nanomaterials provided an additional ∼30% increase, achieving 102 mg/100 mL. Overall, the optimized conditions resulted in a ∼3.9-fold improvement compared to basal media. Early findings demonstrate that MDCK cells exhibit no negative response to the pigment, highlighting its promise for textile dyeing applications. This research demonstrates the successful isolation of an Arctic *Citricoccus*-like strain and systematic optimization of its carotenoid pigment yield, providing preliminary evidence of its biotechnological potential, particularly for textile coloration.

## 1 Introduction

The polar cryosphere region has a high diversity of pigment molecules from microorganisms that inhabit glacier ice cores and marine surface waters, suggesting its role in protecting against environmental stresses (Sajjad et al., 2020). Carotenoid pigments are known to act as a cryoprotective agent and regulate membrane fluidity (Gruszecki and Strzałka, 2005). Microorganisms from cold environments, such as other microbes, attract significant scientific interest due to their non-toxic, biodegradable pigments, which present eco-friendly alternatives for the textile, pharmaceutical, and food industries (Dharmaraj et al., 2009; Prashanthi et al., 2015; Venil et al., 2016a; Chadni et al., 2017; Pasarin and Rovinaru, 2018; Di Salvo et al., 2023). They are also used in the food and feed industries as nutritional supplements for humans and aquaculture feed (Ambati et al., 2014). These pigments can be extracted using conventional (liquid–liquid extraction with organic solvents) and advanced (ultrasound-assisted, microwave-assisted, pressurized liquid, enzyme-assisted, ionic liquid-assisted extraction, and electroporation) techniques (Cajnko et al., 2019; Eleršek et al., 2020; Rajendran et al., 2023). Combining extraction techniques can further improve pigment recovery—for example, mechanical cell disruption followed by solvent extraction yields higher β-carotene recovery from yeast rather than solvent extraction alone (Hladnik et al., 2024).

Owing to their broad industrial applications and relatively simple extraction, researchers have developed a strong interest in isolating high-yield pigment-producing bacteria and developing strategies to enhance pigment output through media optimization and adjustments of physicochemical factors during fermentation, achieving yields that surpass those of wild-type strains (Ram et al., 2020). For instance, *Serratia marcescens* exhibited enhanced prodigiosin yields when pH and aeration were carefully controlled, demonstrating the critical role of environmental factors in pigment biosynthesis (Han et al., 2021). Similarly, the optimization of carbon and nitrogen sources in *Micrococcus luteus* significantly improved carotenoid output, underscoring the importance of medium composition (Dufossé, 2016). To systematically refine these conditions, researchers have employed response surface methodology (RSM) to enhance pigment production. Studies on *Pseudomonas aeruginosa* and *Streptomyces* species have demonstrated that RSM-based modeling can predict optimal combinations of pH, temperature, and nutrient levels, leading to pigment yields far greater than those of wild-type strains (Ram et al., 2020; Joshi et al., 2019).

Building on the optimization framework of physicochemical parameters, recent studies have demonstrated that nanomaterials can act as complementary modulators of the microbial metabolism. Pigment biosynthesis is often a stress response, and nanoparticles at sub-inhibitory concentrations provide controlled stimuli that stabilize or enhance pigment yields. TiO_2_ was selected for its photocatalytic properties, which can stimulate bacterial metabolism under light exposure and have been shown to increase lipid productivity in *Chlorella vulgaris* (Zhao et al., 2022). Fe_3_O_4_ was chosen for its magnetic and redox-active nature, enabling microbial immobilization, electron transfer, and nutrient supply, with evidence of enhanced butyric acid production in *Clostridium tyrobutyricum* when coated with chitosan (Jaber et al., 2023). Together, these nanoparticles serve as reservoirs of micronutrients and scaffolds for microbial growth, complementing physicochemical optimization strategies. Prior reports of improved carotenoid and melanin yields with ZnO, Fe_2_O_3_, and TiO_2_ (Gómez-Gómez et al., 2019; Kang et al., 2014) further support their role as rational enhancers of microbial pigment production.

In this study, we identified a pigmented *Citricoccus*-like bacterium (PS104) from the Arctic fjord of Ny-Ålesund, Svalbard, Norway. Pigment production of PS104 was systematically optimized using one-factor-at-a-time (OFAT) methodology, RSM, and TiO_2_ nanomaterial to maximize yield. The preliminary cytotoxic evolution of the crude pigment extract indicated no adverse effect on MDCK cells. By integrating nanomaterial-assisted optimization with conventional statistical approaches, this study not only reports a novel Arctic pigment-producing isolate but also provides new ecological and methodological insights, with initial evidence of biotechnological relevance in sustainable textile applications.

## 2 Materials and methods

### 2.1 Isolation and growth optimization of pigmented bacteria

Sediment sample was collected from the banks of the Ny-Ålesund fjord, Kongsfjorden, Svalbard, Norway (78.91624° N, 11.95854° E) in sterile containers during the 13th Indian Arctic Expedition. The collected samples were dissolved in sea water, serially diluted (up to 10^−5^), and 100 µL aliquots were spread-plated on 25% Zobell Marine Agar (ZMA) and incubated up to 5 days at 5 ± 2 °C. Tiny colonies appeared at 48 h. The serial dilution plate had 2.3 × 10^-5^ colonies/mL, among which were four to five pigmented colonies. On day 4, isolated colonies were sub-cultured to fresh ZMA plates and stored on 25% ZMA slants at 4 ℃. Samples were transported to Goa University, India at 4 °C in the Indian cargo. In the laboratory, they were checked for growth on nutrient agar (NA) plates at different incubation temperature ranges to consider feasibility, industrial scope, and higher pigment production. Excepting the yellow psychrotrophic isolate PS104, all other isolates showed instability in pigment production and stopped growing upon further culturing. Hence, the PS104 was further studied for pigment production. To investigate the psychrotrophic nature of the isolate, nine Erlenmeyer flasks, each containing 50 mL of nutrient broth, were inoculated with the culture. The flasks were incubated in triplicate at 5, 10, 15, 20, 25, 30, 35, 40, and 45 °C for 5 days, and growth was monitored by measuring O.D. at 600 nm.

### 2.2 Identification and characterization of pigmented bacteria

We performed Gram staining of bacterial isolate using a Gram Staining Kit (K001, HiMedia, India) to confirm Gram characteristics (Coico, 1997). Cell morphology and size were examined by scanning electron microscopy (SEM) (Evo 18, Carl Zeiss, Germany) (Bratbak, 2018). We performed biochemical characterization of isolates for SIM (sulphur, indole, motility), MR-VP (Methyl Red–Voges Proskauer tests), citrate utilization, urease, gelatinase, nitrate reduction, ONPG (ortho-nitrophenyl-β-galactoside), oxidase, and catalase tests. To further validate the bacterium’s identity, we performed molecular identification by partial sequencing of the 16S rRNA gene. The sequence was analyzed using BLASTn against the NR database, and a phylogenetic tree was constructed in MEGA X using the neighbor-joining method with 1,000 bootstrap replicates. The detailed methodology is available in the supplementary methods section.

### 2.3 Pigment production, maturation, and characterization

#### 2.3.1 Crude pigment extraction

The culture was grown in 100 mL nutrient broth at 25 °C on a rotary shaker at 100 RPM for 5 days. Solvent extraction was carried out using polar solvents such as ethanol, methanol, and acetone and non-polar solvents such as hexane, chloroform, and diethyl ether. For each solvent, 10 mL of 5-day old culture was centrifuged at 10,000 RPM for 15 min at 4 °C. The collected pellet was washed twice with distilled water and re-suspended in 5 mL solvents, vortexed, and kept overnight at 4 °C. On the subsequent day, the supernatant containing colored pigment was collected by centrifugation at 10,000 RPM. The absorption spectrum of this supernatant was recorded using a UV–visible spectrophotometer. The extracted pigment was stored at 4 °C for subsequent experiments (Chaturwedi et al., 2024). The stability of the pigment was evaluated with respect to pH, temperature, and UV exposure. For pH stability, 1.0 mL aliquots of the pigment solution were adjusted from pH 2 to 14. For thermal stability, pigment solution at 20, 30, 40, and 50 °C was incubated for 15 min in a water bath at a set temperature. UV stability was determined by exposing 4 mL pigment aliquots in a closed UV cabinet (250 ± 10 nm) for 0, 5, 10, 15, and 30 min. The absorption spectrum of each sample was recorded against untreated controls. Detailed methodology is available in the supplementary methods section.

#### 2.3.2 Fourier transform infrared spectroscopy and high-resolution liquid chromatography mass spectrophotometry

The crude methanolic pigmented extract was dried and subjected to silica gel column chromatography (60–120 mesh) for purification. Sequential elution was performed using solvents of increasing polarity with hexane, followed by hexane–ethyl acetate (1:1, v/v), pure ethyl acetate, ethyl acetate–methanol (1:1, v/v), and methanol. The flow rate was maintained at 1.1 mL/min, and the pigmented elute was collected in a sterile vial (Poddar et al., 2021). The pigmented fraction was analyzed by Fourier transform infrared (FT-IR) spectroscopy (Shimadzu IR Prestige-21) (Simsek Geyik, et al., 2025) and high-resolution liquid chromatography mass spectrophotometry (HR-LCMS) (QTOF, Agilent Technologies, United States). For FT-IR, the sample was dried on infrared-transparent salt plates to remove solvent and form a thin dry film of the sample. The transmittance spectrum was recorded over the range of 4,000 to 400 cm^-1^. HR-LCMS was performed to analyze potential metabolites present in the pigmented sample (Joglekar and Suruchi, 2023). Chromatographic separation was achieved on a ZORBAX Eclipse Plus C18 column (150 × 2.1 mm, 5 μm; Agilent), with 0.1% formic acid in Milli-Q water and acetonitrile as the mobile phase. Compounds were identified via their mass spectra and their unique mass fragmentation patterns. Data was acquired and processed by MassHunter Qualitative Analysis B.06.

#### 2.3.3 Time-dependent pigment maturation

We performed the time dependent pigment maturation to ascertain the incubation time required for the complete maturation of pigment. A set of six flasks containing 50 mL of nutrient broth were inoculated with 1.0 mL starter culture and incubated in a rotary shaker at 100 RPM at 25 °C. After 24 h, one flask was used to extract the pigment. The procedure was continued for 6 days and the absorbance spectra of the pigment were determined each day. The maximum absorbance at respective λ_max_ was recorded as the pigment maturation time.

### 2.4 Optimization of pigment production

Freshly grown culture was inoculated in 100 mL basal medium and incubated for 3 days on a rotary shaker, 100 RPM at 25 °C (Supplementary Table S1). The pigment was extracted followed by incubation and dried by evaporation of the methanol solvent. The dry weight of the pigment was measured, and its yield was recorded. The experiment was conducted in triplicate; data are expressed as mean values with standard deviations (±SD).

#### 2.4.1 Screening one-factor-at-a-time for pigment production

We further studied carbon-, nitrogen-, salt-, pH-, and temperature-dependent pigment production using the above growth conditions (Elkenawy et al., 2017; Srimathi et al., 2017). One-way ANOVA was employed to compare the efficacy of different carbon and nitrogen sources and to determine the statistical significance. Centered second-order quadratic polynomial models and nonlinear regression analyses were applied to elucidate the relationships between carbon and nitrogen concentrations, salt concentration, pH, temperature, and pigment dry weight (*p* < 0.05). The detailed methodology for each factor is shown in the supplementary methods. Statistical analyses were carried out using GraphPad Prism, with experiments conducted in triplicate; data are expressed as mean values with standard deviations (±SD).

#### 2.4.2 Response surface methodology analysis

RSM was applied using Design Expert software, version 11.1.2.0 (Stat-Ease Inc., Minneapolis, MN, United States). Physical and chemical growth factors defining the pigment production, such as carbon source, nitrogen source, sodium chloride concentration, and incubation temperature, were considered for analysis. The optimal conditions for improving pigment yield were performed using a randomized Box–Behnken RSM design. Factor levels were coded as −1 (low) and +1 (high) (Venil et al., 2015). The detailed experimental design of the model used is shown in the supplementary methods.

#### 2.4.3 Nanomaterial-assisted pigment yield enhancement

We tested nanomaterial-assisted pigment yield using RSM medium supplemented with commercially procured nanomaterials such as TiO_2_ (40566, CAS: 13463-67-7, SRL, India) with average particle size (APS) 250 nm and purity 99.7%, and Fe_3_O_4_ (16708, CAS: 1317-61-9, SRL, India), with APS 20–50 nm and purity 99.9%. Both TiO_2_ and Fe_3_O_4_ were used with varying concentrations of 25, 50, 100, and 200 μg/mL. Nanoparticles were dispersed in MilliQ water and subjected to pulsed sonication using a probe sonicator (SONIC-650WT-V2, MRC LTD., Hagavish 3 St. Holon, 58817, Israel) for 25 min. The experiment was conducted in triplicate; the pigment dry weight is expressed as mean values with standard deviations (±SD).

We also confirmed the cytotoxic effect of nanoparticles on the bacteria by flow cytometry. For this, cells were harvested at 9500 RPM from 1.0 mL RSM medium supplemented with 25 μg/mL and 200 μg/mL TiO_2_ or Fe_3_O_4_. The pellets were washed with sterile phosphate-buffered saline (PBS), resuspended, and the number was fixed to 10^6^ cells/mL. The samples were gently mixed and incubated with propidium iodide (PI) (10 μg/mL) for 5 min at room temperature in the dark. All samples for live/dead assays were evaluated using flow cytometry (FACSMelody cell sorter, BD Biosciences, United States) (Yamagami et al., 1994). A 488 nm laser was used for excitation. PI fluorescence was recorded using a 685 nm long-pass mirror and bandpass filter with 695/40 nm transmission. Gating was employed in a PI-stained population v/s unstained control, considering the average mean value of control versus PI-stained population to avoid selecting a weak signal. Data acquisition and analysis were performed using BD FACSChorus software.

### 2.5 Early evaluation for biotechnological potential of bacterial pigments

#### 2.5.1 Evaluation of antioxidant and dyeing applications

We performed a DPPH (2,2-diphenyl-1-picrylhydrazyl) assay to check the potential of pigment extract as an anti-oxidant agent (Venil et al., 2016b). Ascorbic acid was used as a control. To evaluate the potential of the pigment as a textile dye, seven commercially available fabrics—cotton, jute, poplin, dacron, polyester, terry cotton, and viscose—were selected for dye retention capacity with four different types of mordants: acidic (0.05% (w/v) acetic acid), alkali (0.05% (w/v) sodium bicarbonate), metal (0.05% (w/v) copper sulfate), and salt (0.05% (w/v) sodium chloride) (Morales-Oyervides et al., 2017; Krishna et al., 2008). The details of the experiments are available in the supplementary methods.

#### 2.5.2 Cell cytotoxicity assay

Madin–Darby Canine Kidney (MDCK) cells were cultured in Dulbecco’s modified Eagle medium (DMEM) (10% fetal bovine serum (FBS) and 1% antibiotics-antimycotic with 10,000 unit/mL of penicillin G, 10 mg/mL of streptomycin) solution at 37 °C in a humidified environment with 5% CO_2_. Cells were treated with 5 µL concentrated pigment extract (4 µg/5 µL) in 2 mL medium for 72 h. Cells were harvested by trypsinization (1X trypsin–EDTA Solution, HiMedia), stained with 10 μg/mL PI for 10 min, washed twice, and imaged under fluorescent microscopy for cell cytotoxicity (Yamagami et al., 1994). Cell viability was assessed by quantifying total and stained cells across ten randomly selected fields of view, with values averaged and represented in the viability graph.

### 2.6 Statistical analysis

All statistical analyses were performed using GraphPad Prism version 11.0.0 (GraphPad Software, Boston, MA, United States) or Design-Expert version 11.1.2.0 (Stat-Ease, Minneapolis, MN, United States). RSM was conducted using Design-Expert, and model adequacy was validated by analysis of variance (ANOVA). For statistical analysis, 27 runs with 24 mid-points and 3 replications of the central point were selected. Optimization of the pigment yield was carried out using a randomized Box–Behnken design, with factor levels coded as −1 (low) and +1 (high). The details are available in the supplementary methods.

For statistical analyses of OFAT, pigment yield, and other data, GraphPad Prism was used for general data evaluation. One-way ANOVA was applied to compare pigment yields across different carbon and nitrogen sources. Centered second-order quadratic polynomial models and nonlinear regression analyses were employed to examine the effects of carbon source, nitrogen source, salt concentration, pH, and temperature on pigment dry weight. Wherever applicable, experiments were conducted in triplicate; data are presented as mean values ± ±SD. Statistical significance was determined and presented in the data at *p* > 0.05 (ns). *p* < 0.05 (*), *p* < 0.01 (**), *p* < 0.001 (***), and *p* < 0.0001 (****).

## 3 Results and discussion

### 3.1 Isolation of pigment-producing bacteria from the Arctic fjord

The Arctic sample plates were incubated at 5 ± 2 °C and monitored daily for 5 days. After 2 days, faint growth of non-pigmented pinpoint colonies became visible. By the end of the five-day incubation period at 5 ± 2 °C, several fully developed colonies were observed, comprising both pigmented and non-pigmented bacteria. We selected four pigmented isolates and maintained them on nutrient agar at 5 ± 2 °C. When sub-cultured and maintained at 25 °C, only isolate PS104 consistently exhibited stable pigment production, reflecting its psychrotrophic nature. PS104 was growing well from 5–30 °C (Figure 1). This isolate was sub-cultured and maintained at −80 °C in a 55% glycerol stock for further experiments.

**FIGURE 1:**
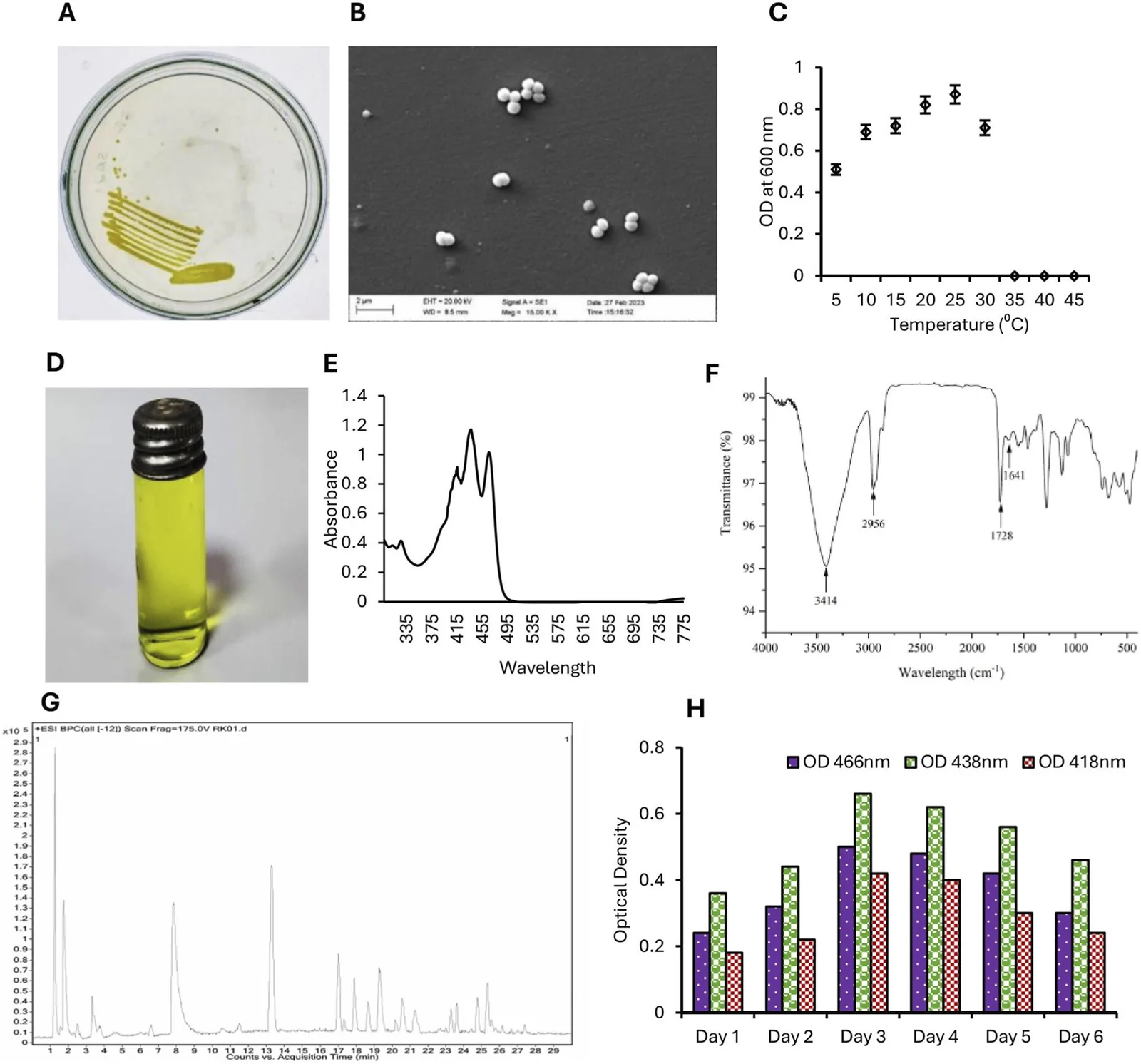
Characteristics of PS104 bacteria and its pigmented crude extract. **(A)** Growth of PS104 on nutrient agar. **(B)** SEM image of PS104 bacterial cells. **(C)** Temperature-dependent growth of PS104. **(D)** Yellow pigmented methanolic extract of PS104. **(E)** UV–visible spectrum of the pigment with three absorption peaks at 466 nm, 438 nm, and 416 nm. **(F)** FT-IR spectrum of the yellow pigmented fraction. **(G)** HR-LCMS spectra of the yellow pigmented fraction. Table S-3 lists all known compounds and pigment-contributing yellow color identified. **(H)** Time-dependent-pigment maturation.

Previous studies have reported yellow pigment-producing γ-proteobacteria from Antarctica (Poli et al., 2007; Hwang et al., 2009; Koh et al., 2013). More recent research has identified yellow carotenoid pigments in psychrotolerant bacteria such as *Chryseobacterium* from Antarctic soils (Órdenes-Aenishanslins et al., 2016; Marizcurrena et al., 2019). Pigment production in the Arctic has also been reported: Sinha et al. (2017) described a quinolone-based yellow pigment produced by α-proteobacteria, while Stafsnes et al. (2010) identified yellow carotenoid-producing bacteria along the Norwegian coast. Although *Citricoccus* species have been documented in mold-colonized walls, human skin, and desert soils (Li et al., 2005; Schäfer et al., 2010; Ndiaye et al., 2018), their recovery from Arctic waters provides definitive evidence of their survival in the extreme cold there. Our finding therefore revised the current understanding of microbial resilience in polar habitats and represents a significant expansion of current ecological knowledge. Collectively, these studies underscore the widespread occurrence and ecological significance of yellow pigment-producing bacteria across both polar and subpolar environments.

### 3.2 Characterization of the pigment-producing bacteria

#### 3.2.1 Morphological and biochemical assays

The PS104 colony showed a bright yellow appearance, entire margin, and smooth texture with raised elevation on nutrient agar (Figure 1A). Gram staining of the culture revealed Gram-positive cells with cocci morphology. SEM images revealed a spherical cell morphology with a diameter of 0.64 µm (Figure 1B). Biochemical characterization study confirmed that the PS104 strain demonstrated an ability to produce acetoin, utilize citrate as its sole carbon source, and produce oxidase and catalase enzymes. These capabilities were confirmed through positive in Voges–Proskauer test results for acetoin production, the citrate utilization test for carbon source utilization, and enzyme activity tests for the presence of oxidase and catalase enzymes (Supplementary Figure S1; Supplementary Table S2).

#### 3.2.2 Molecular identification

We performed BLASTn analysis of the 16S rRNA gene of the isolated strain PS104. Our results indicate a close phylogenetic relationship between the isolated strain and various species of the Micrococcaceae family, with 98.99% similarity to *Citricoccus parietis* strain 02-Je-010. Similarly, the biochemical characterization of *C. parietis* strain 02-Je-010 indicates catalase- and oxidase-positive reactions (Schäfer et al., 2010). These results are consistent with those reported for strain PS104. Furthermore, positive acetoin production and catalase utilization further supports the metabolic profile of Micrococcaceae members, although these traits can vary across genera. The biochemical assay validation with the 16S rRNA sequence similar to *C. parietis* supports isolate PS104 being related to the Micrococcaceae family. Specifically, the catalase- and oxidase-positive reactions are hallmark traits of *C. parietis*, and their presence in PS104 validates the 16S rRNA-based phylogenetic placement. Another closely related bacterial strain, *C. zhacaiensis* FS24, supports isolate PS104 being a member of Micrococcaceae (Meng et al., 2010). The taxonomic position of the Arctic bacteria in the phylogenetic tree along with their closest relatives is illustrated in Supplementary Figure S2. The nucleotide sequence was deposited in GenBank, accession number OQ257376.

Members of this family are predominantly isolated from mammalian diverse environmental sources (Dastager et al., 2014). Several members, particularly *M. luteus*, *M. lylae*, and *M. roseus*, are notable pigment producers, commonly yielding yellow to orange pigments (Benjamin et al., 2016; Shahin et al., 2022; Kandasamy and Kathirvel, 2024). Previous research has reported the presence of *Citricoccus* species in diverse habitats, including the desert soil of Egypt (Li et al., 2005), the Cuatro Cienegas Basin in Coahuila, Mexico (Hayano-Kanashiro et al., 2011), soil samples from the Ryukyu Archipelago, Japan (Matsui et al., 2012), and marine macroalgae from Antarctica (Leiva et al., 2015). *Citricoccus* species from non-Arctic environments have also been reported for synthesizing gold nanoparticles and for their antimicrobial activity (Camas et al., 2019; Pérez Matos et al., 2023). Despite previous explorations, the present study provides a pigment-producing bacterium from a new ecological niche, closely related to *Citricoccus* species, which was isolated from an Arctic fjord. However, whole-genome sequencing and average nucleotide identity (ANI) analysis would be required for precise taxonomic assignment.

#### 3.2.3 Determining the psychrotrophic nature of PS104

To classify thermal preferences into psychrophilic, psychrotrophic, or mesophilic, a temperature gradient spanning the typical ranges for each category was employed for PS104. It exhibited growth and pigment production at 5–30 °C, with no growth above 30 °C (Figure 1C). This adaptability across a wide temperature range suggests that PS104 is psychrotrophic in nature. Psychrophilic organisms are defined by optimal growth below 15 °C, whereas psychrotrophic organisms grow at 0–30 °C (Gounot, 1986).

### 3.3 Spectral analysis of PS104-derived extract

The pigment synthesized by the Arctic bacterium was intracellular, with no extracellular secretion detected in the culture medium. Solubility analysis confirmed its polar nature, with successful extraction only in polar solvents (e.g., methanol, ethanol) and no dissolution in non-polar solvents. PS104 released its yellow pigment in methanol; thus, among the polar solvents, methanol revealed an optimal extraction efficiency yielding a colorless cell pellet (post-extraction) (Figure 1D). Subsequent to centrifugation, the supernatant was subjected to UV–visible spectrophotometric analysis, revealing a three-shoulder absorption spectrum with λ_max_ at 466 nm, 438 nm, and 416 nm—a spectral signature consistent with carotenoid pigments, which typically exhibit λ_max_ of 400–500 nm (Figure 1E) (Lichtenthaler and Buschmann, 2001). The pigment’s stability was evaluated under varying pH, temperatures, and shortwave UV exposures (Supplementary Figure S3). The pigment remained spectrally stable between pH 2.0 and pH 12.0; however, the spectral shift was observed at highly alkaline conditions (pH > 12.0), indicating possible degradation. Thermal stability testing showed no significant alteration of the absorption spectrum after 15 min incubation at 20, 30, 40, or 50 °C, indicating stability across this temperature range. In contrast, exposure to UV light resulted in progressive spectral loss as exposure times increased from 5 to 20 min.

### 3.4 FT-IR and HR-LCMS analysis of the crude pigment extract

Column chromatography, employing an ethyl acetate and methanol (1:1, v/v) mixture, resolved the extract into a distinct pigmented fraction. FT-IR spectra of the pigmented fraction show a sharp broad band at 3,414 cm^-1^, a narrow peak at 2,956 cm^-1^, a sharp narrow band at 1,728 cm^-1^, corresponding to O-H, C-H, C=O stretching, respectively. A minor band observed at 1,641 cm^-1^ resembles C=C stretching (Figure 1F).

Chromatographic separation of high-resolution LCMS analysis under positive ionization conditions revealed multiple peaks between 1.2 and 25.6 min, corresponding to diverse metabolites and pigment-related compounds, with analytes detected across a mass-to-charge (m/z) range of 98–568 (Figure 1G). The pigmented fraction was seen to consist of many metabolites, amongst which many are known to appear yellow, including N-nitrosopyrrolidine, trimethyloxazole, 5,6,7,8-tetrahydro-2,4-dimethylquinoline, 2,4-diethyl-5-propylthiazole, benfluralin, 2-(3-phenylpropyl)pyridine, 5-hydroxy-4-pentenoic acid d-lactone, and oleamide. Among these, a distinct peak detected at 22.19 min with a m/z value of 568.431 was putatively identified as (3S, 3′S,all-E)-zeaxanthin (C_40_H_56_O_2_) based on MS/MS fragmentation matching. The compound exhibited a mass error of −5.18 ppm, confirming high-precision mass accuracy. Database matching (compound table hits = 10) and molecular formula consistency further supported this identification. These results indicate that the coloration of the culture arises from the combined effect of carotenoid-like pigments, associated metabolites, and additional unidentified compounds (Supplementary Table S3). Taken together with column chromatography evidence, these findings substantiate the pigment-producing capability of the PS104 isolate.

### 3.5 Time-dependent pigment maturation

Temporal analysis of pigment production from the PS104 strain indicated the continuous biosynthesis of the pigment from days 1 to 6, exhibiting a gradual increase in optical density until day 3, followed by a gradual decline (Figure 1H). This daily assessment indicated a pigment maturation peak on day 3.

### 3.6 Optimization of pigment production through the OFAT method

Our experimental results indicated 26 mg/100 mL pigment production from PS104 in basal medium at pH 7 and 25 °C incubation. Modification of the basal medium composition, along with the adjustment of pH and incubation temperature, resulted in an increased dry weight of the crude pigment produced. Specifically, glycerol-enriched media produced 1.4-fold higher pigment yields than glucose-containing media (Figure 2A). One-way ANOVA revealed that then carbon source had a highly significant effect on pigment production (F (8, n–9) = 811.9, *p* < 0.0001, R^2^ = 0.997). Tukey’s HSD *post hoc* test confirmed that glycerol supplementation was significantly more effective than glucose (ΔM = −8.33, 95% CI [−10.07, −6.59], q = 23.7, *p* < 0.0001). These results demonstrate that glycerol supported preferentially increased pigment production, consistent with previous reports of enhanced red pigment synthesis in *Monascus ruber*, when glycerol was used as a carbon source (Meinicke et al., 2012). In contrast, lactose yielded minimal pigment, while starch failed to support growth (Supplementary Table S4).

**FIGURE 2:**
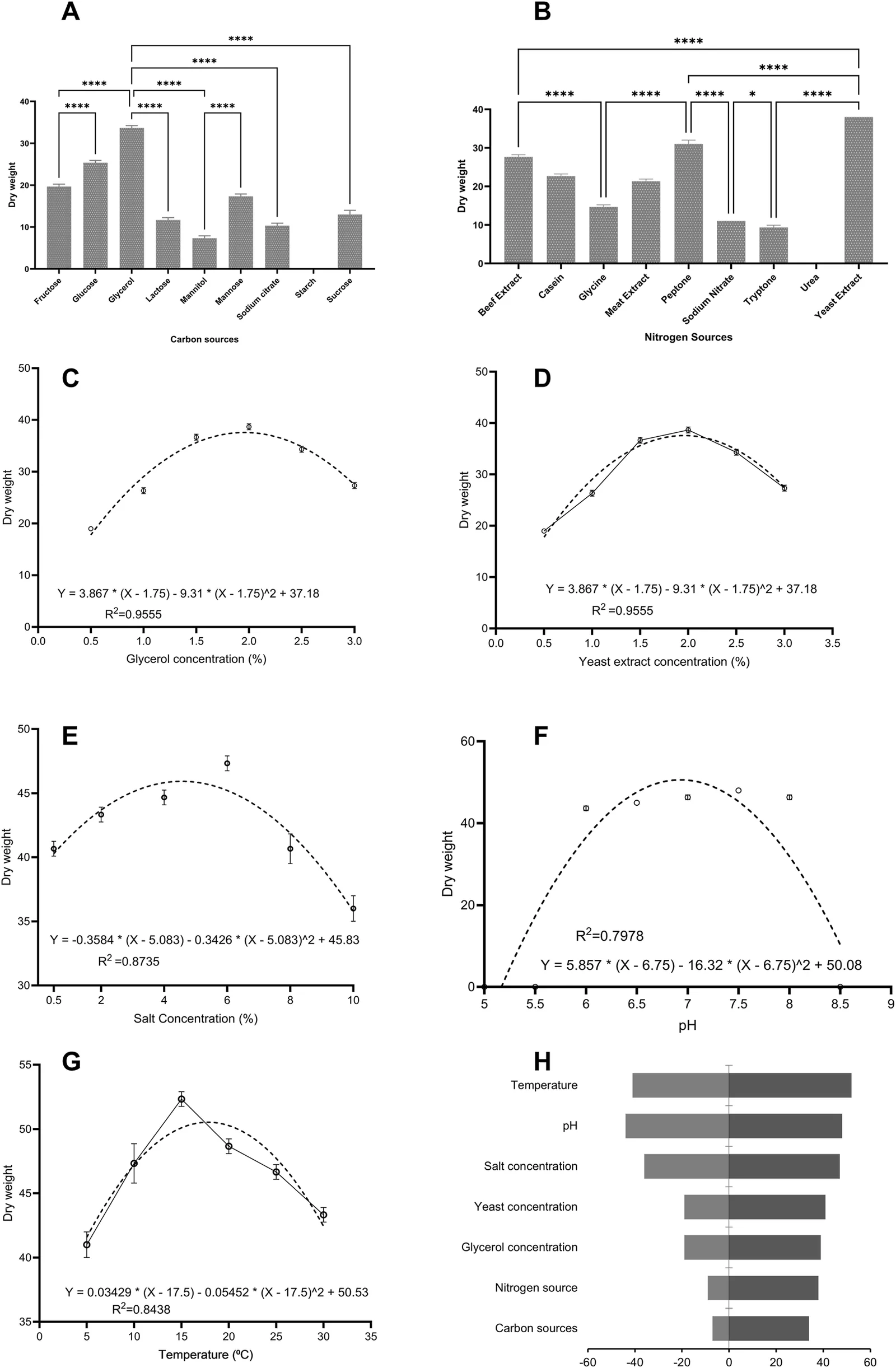
Optimization of pigment production using OFAT analysis. **(A)** Effect of different carbon sources on pigment production, with glycerol supporting maximum yield. One-way ANOVA with Tukey’s HSD *post hoc* test showed a highly significant overall effect of carbon source on pigment production (*p* < 0.0001). **(B)** Effect of different nitrogen sources, where yeast extract resulted in the highest pigment production. One-way ANOVA followed by Tukey’s HSD *post hoc* test revealed a highly significant effect of nitrogen source on pigment production (*p* < 0.0001). **(C–G)** Effects of glycerol concentration, yeast extract concentration, NaCl concentration, pH, and incubation temperature on pigment production, respectively. Quadratic regression models showed good agreement with experimental data (*R* ^2^ values: 0.9555, 0.9555, 0.8735, 0.7978, and 0.8438, respectively). **(H)** Tornado chart plotted from A–G, showing the relative influence of different factors on pigment production. n = 3, statistical significance is indicated by asterisks.

Pigment production also varied significantly with nitrogen source: yeast extract yielded the highest pigment levels, followed by peptone and beef extract, whereas urea supported no growth (Figure 2B). One-way ANOVA confirmed a highly significant effect of nitrogen source on pigment production (F (8, n-9) = 1,435, *p* < 0.0001, *R* ^2^ = 0.998), significantly greater than peptone (ΔM = −7.00, 95% CI [−8.56, −5.44], q = 22.28, *p* < 0.0001) and beef extract (ΔM = −10.33, 95% CI [−11.89, −8.78], q = 32.88, *p* < 0.0001), while urea supported no growth (Supplementary Table S5). Our findings revealed that the medium composed of 2% glycerol, 1.5% yeast extract, and 6% sodium chloride at pH 7.5 and an incubation temperature of 15 °C showed the highest pigment production of 52 mg/100 mL (Figures 2C–G). Centered second-order quadratic polynomial models and nonlinear regression analyses showed agreement with the experimental data, with coefficient of determination (R^2^) values of 0.9555, 0.9555, 0.8735, 0.7978, and 0.8438, respectively, indicating that the design captured most of the variability in pigment production. The strong model fits for several responses suggest that the relationships between medium components and pigment yield are predominantly quadratic rather than strictly linear. A tornado chart highlights the relative influence of different factors on pigment production, with temperature exerting the highest positive effect, followed by pH, salt concentration, yeast extract concentration, glycerol concentration, nitrogen source, and carbon source (Figure 2H).

### 3.7 Box–Behnken design for response surface methodology analysis

Box–Behnken design (BBD) was employed to assess interactions among significant factors such as concentration of glycerol (Factor A), yeast extract (Factor B), NaCl (Factor C), and temperature of incubation (Factor D) and also to determine the best combination of factors to obtain highest dry weight of the produced pigment. Parameters with coded high and low values were set for each factor. For statistical analysis, a total of 27 runs with 24 mid-points and 3 replications of the central point were selected. Supplementary Table S6 presents both experimental and predicted values of the response obtained for all 27 runs. The responses ranged 14–74 mg, yielding a maximum-to-minimum ratio of 5.3. Since this ratio was below 10, no data transformation was required.

The outcome of the mathematical models was validated through experimental data and analyzed through regression analysis. The significance of each coefficient is listed in Supplementary Table S8. ANOVA showed significance with an F-value of 26.18, overall *p*-value of <0.0001, and a lack-of-fit test value of *p* = 0.0566). The lack-of-fit test (F = 17.07) suggested that the model possesses strong predictive capability. The fitness model gives R^2^ = 0.9683, which showed strong prediction response. The predicted R^2^ (0.8187) is in reasonable agreement with adjusted R^2^ (0.9313) due to the small difference (0.2) between the two (Supplementary table S7). The model-fitting results indicated that three linear terms (A, C, and D), three interaction terms (AB, AC, and AD), and four quadratic terms (A^2^, B^2^, C^2^, and D^2^) were statistically significant (*p* ≤ 0.05). Exact *p*-values for model terms are reported in Supplementary Table S8.

The accuracy of the implemented model was confirmed from the generated diagnostic plot of experimental and model outcomes (Figures 3A–F). Figure 3A shows that residuals followed a normal distribution, with points closely aligned to the straight line and no variance deviation, confirming the model’s adequacy in capturing the relationship between process variables and response. In Figure 3B, the random distribution of residuals around the zero line without any distinct pattern assures homoscedasticity and the absence of systematic error in the model predictions. The residuals-versus-run order plot illustrates the random scatter of residual values across experimental runs, indicating the independence of observations and absence of time-dependent variation or experimental bias (Figure 3C). The predicted-versus-actual response plot compares experimentally observed dry weight values with model-predicted values (Figure 3D). The close alignment of data points along the diagonal line demonstrates strong agreement between predicted and actual responses and confirms the reliability of the regression model. The Box–Cox plot for power transformation presents the relationship between lambda (λ) values and the residual sum of squares (Figure 3E). The perturbation plot revealed that pigment yield was highly sensitive to variations in specific factors, as indicated by the steep curvature of their response lines (Figure 3F). All variables exhibited quadratic behavior, confirming the presence of optimal levels within the experimental range. The contour plot shows the cumulative effect of the independent variables on the dry weight of the pigment. If the contour is elliptical, then mutual combination of the variables is more prominent in determining the response, whereas circular contours indicate that the variables when present in combination might be less prominent in affecting the response (Figures 3G–L) (Tanyildizi et al., 2005). A three-dimensional response-surface curve determines the interaction effects and ideal value of the variables, which will lead to a higher response. The maximum pigment yield was observed near the central point of the design space, while extreme levels of factors resulted in a decline in production (Supplementary Figure S4). Responses included the dry weight of pigment increasing with increased carbon concentration, nitrogen concentration, NaCl concentration, and temperature to a particular point then further show a steady decline.

**FIGURE 3:**
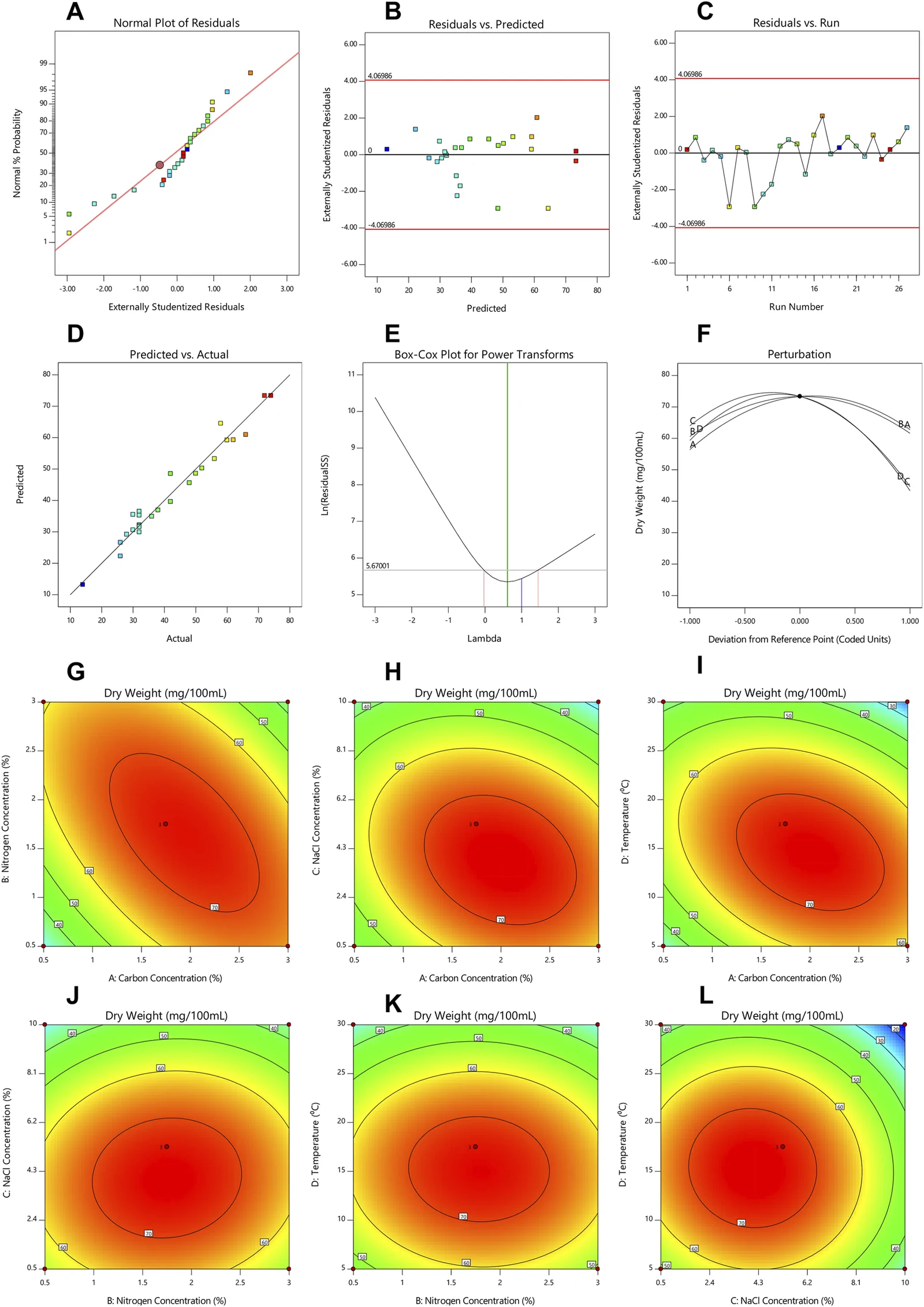
Statistical validation and response surface analysis for the optimization of pigment production (expressed as dry weight, mg/100 mL) using RSM. **(A)** Normal probability plot of externally studentized residuals showing the residuals distributed approximately along a straight line, indicating normal distribution of experimental errors and adequacy of the developed quadratic model. **(B)** Plot of externally studentized residuals vs. predicted response values. **(C)** Residuals vs. run order plot illustrating the random scatter of residual values across experimental runs **(D)** Predicted vs. actual response plot comparing experimentally observed dry weight values with model-predicted values. **(E)** Box–Cox plot for power transformation showing relationship between lambda (λ) values and residual sum of squares. Optimum λ value close to 1 indicates no significant transformation of response data. **(F)** Perturbation plot illustrating the individual effect of coded process variables on dry weight production while keeping other variables constant at the reference point. **(G-L)** Two-dimensional contour plot showing the interactive effect of **(G)** carbon and nitrogen, **(H)** carbon and NaCl, **(I)** carbon and incubation temperature, **(J)** nitrogen and NaCl, **(K)** nitrogen and incubation temperature, **(L)** and NaCl and incubation temperature on dry weight production.

The highest pigment yield of 76.7 mg/100 mL was suggested by the model with the factors 2.3% carbon concentration, 1.4% nitrogen concentration, 3.6% NaCl concentration, and 14 °C incubation temperature. The model successfully identified the key factors influencing pigment yield as the response obtained with the optimized medium composition with a 1.5-fold increase in pigment yield of 78 mg/100 mL (Figure 4A). Overall, the application of RSM resulted in a threefold enhancement of pigment yield. Detailed statistical analysis is available in the supplementary results.

**FIGURE 4:**
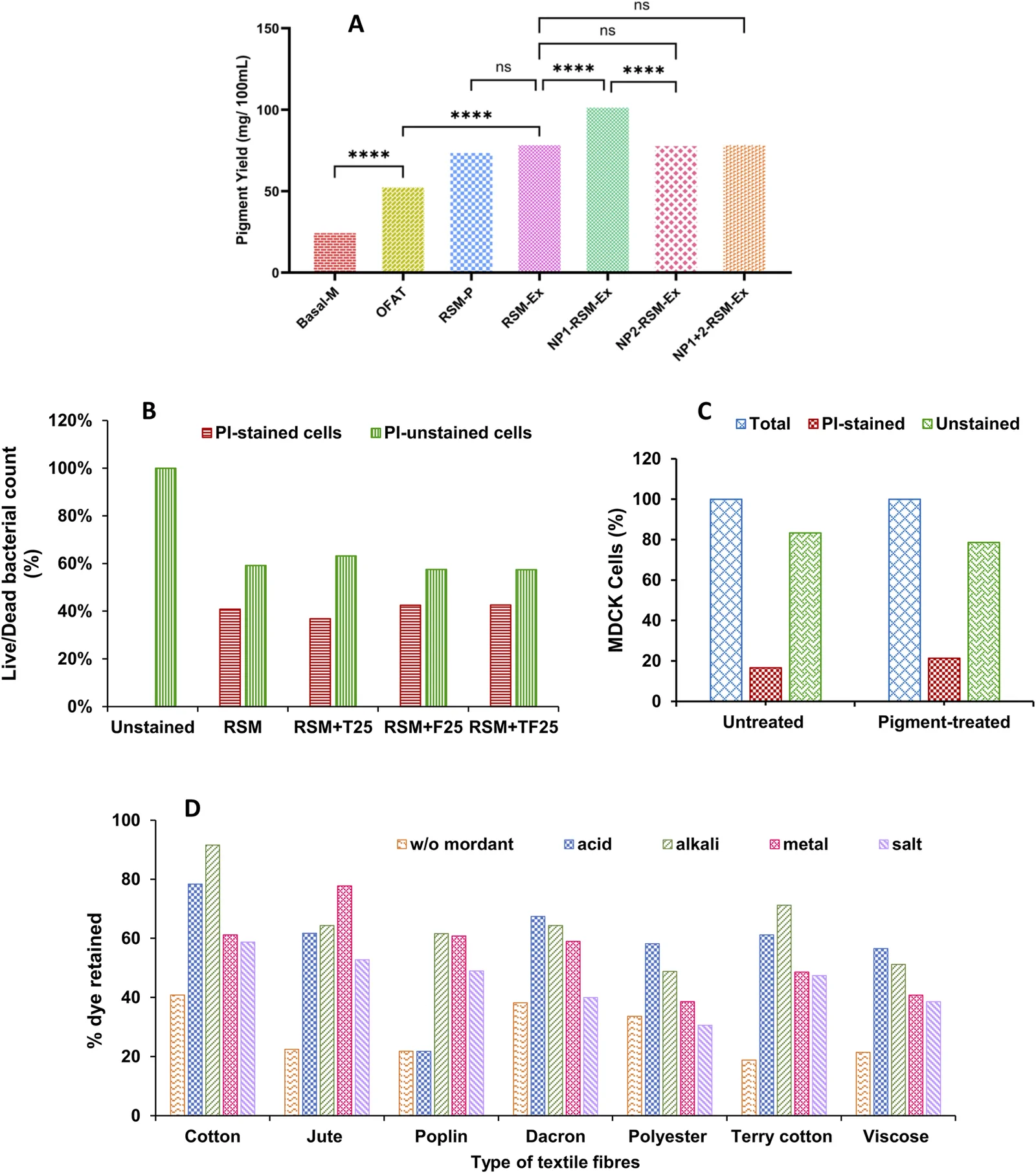
Optimization of pigment yield, cytotoxicity, and dying applications of the crude pigment. **(A)** Pigment yield across different media formulations. One-way ANOVA indicated a significant effect of medium composition on pigment production (*p* < 0.0001). Significant differences between treatments were determined by Tukey’s HSD test. **(B)** Live/dead cell count using flow cytometry with propidium iodide staining. PI fluorescence is recorded with bandpass filter of 695/40 nm transmission. **(C)** Cell toxicity for control-untreated and pigment-treated MDCK cells. **(D)** Dye retention capacity of fabrics with different mordants. NP1: 25 μg/mL TiO_2_. NP2: 25 μg/mL Fe_3_O_4_. OFAT, one-factor-at-a-time. RSM-P, response surface methodology-predicted. RSM-Ex, response surface methodology-experimental. PI, propidium iodide. Statistical significance indicated by asterisks.

### 3.8 Nanomaterial assisted pigment yield enhancement

A further increase of approximately 1.3-fold (ΔM = +23 mg/100 mL; 95% CI [15.9, 29.9]) in pigment production was observed when the RSM-optimized medium was supplemented with 25 μg/mL TiO_2_ nanoparticles, yielding 102 mg/100 mL. In contrast, Fe_3_O_4_ supplementation produced yields comparable to the RSM medium (Figure 4A; Supplementary Table S9; Supplementary Figure S5). One-way ANOVA confirmed that the medium composition significantly influenced pigment production (*p* < 0.0001), and Tukey’s HSD *post hoc* analysis revealed significant differences between basal, OFAT, and RSM media (*p* < 0.0001). Experimental and predicted yields of the optimized RSM medium did not differ significantly (*p* = 0.2829; q = 3.339), validating the model’s predictive accuracy. Pigment yields from the RSM medium were statistically indistinguishable from those obtained with Fe_3_O_4_ (*p* > 0.9999; q = 0.414) or TiO_2_+Fe_3_O_4_ combinations (*p* > 0.9999; q = 0.1151), but significantly higher with TiO_2_ supplementation alone (*p* < 0.0001, q = 15.84). Collectively, these findings highlight the critical role of medium composition and nanoparticle supplementation, with TiO_2_ exerting a pronounced positive effect on pigment production.

Previous studies have demonstrated that nanoparticles such as Ag/TiO_2_ may either inhibit or stimulate pigment biosynthesis depending on their concentration, physicochemical properties, and interaction with microbial metabolism (Gómez-Gómez et al., 2019; Jaber et al., 2023). In our study, supplementation of the RSM-optimized medium with TiO_2_ nanoparticles at 25 μg/mL enhanced pigment yield by ∼1.3-fold, suggesting a stimulatory effect under controlled conditions. There could be multiple possible mechanisms, including the induction of mild stress responses, modulation of redox homeostasis, membrane permeability, and changes in the local availability of precursors or metal ions (Koul et al., 2021). Further studies under controlled light/dark conditions with variable nanoparticle concentrations will help establish the photo-modulatory effect of nanomaterials on pigment yield.

To confirm the modulatory effects of nanoparticles without severely compromising cell viability, live/dead cell counts were performed using flow cytometry on PS104 cells treated with TiO_2_ and Fe_3_O_4_ nanoparticles. Unstained bacterial cells were used as the negative control for establishing baseline fluorescence. The bacterial cells treated with nanoparticles exhibited similar population distributions to the control cells (Figure 4B). There were no significant changes in numbers of bacterial cells or major shifts in FSC/SSC profiles observed, indicating that both TiO_2_ and Fe_3_O_4_ nanoparticles did not exert a prominent cytotoxic effect on PS104 cell viability. Slight variations in PE fluorescence intensity may result from nanoparticle interaction with bacterial pigments or minor physiological responses (Figure 4B; Supplementary Figure S6).

### 3.9 Cell cytotoxicity study

The cells recorded in the fluorescent image taken from 10 different fields of view at a ×10 objective lens were totaled individually in both untreated control and treated cells with pigments (Figure 4C). In pigment-treated samples, ∼20% cells were PI-stained compared to the untreated control with ∼17% cells. This was expected as the cells were growing for 4 days in the same culture plates. This evaluation in the tested conditions suggests no observable difference in cell viability between control-untreated and pigment-treated cells, suggesting that the pigment may be suitable for textile and other applications. To comprehensively assess the cytotoxic effects, future *in vivo* study is required to validate the pigment’s biocompatible nature.

### 3.10 Antioxidant activity

Standard dilutions (1–10 μg/mL) of ascorbic acid showed 11.11%–89.22% radical scavenging activity (RSA) (Supplementary Table S10). Absorbance of the pigment sample was found to be 0.069, which presented RSA as 86.22%. Our preliminary results support the antioxidant activity of the pigment sample.

### 3.11 Mordant-assisted dyeing potential of textile

Dye retention (%) varied according to mordant type and fabric category (Figure 4D; Supplementary Figure S7). Among natural fibers, cotton and poplin exhibited the highest dye retention when treated with alkali mordant, whereas jute showed peak retention with metal mordants. Synthetic fibers, including dacron and polyester, showed superior retention with acid mordants. For semi-synthetic fibers, alkali mordants yielded optimal results on terry cotton, while acid mordants performed best on viscose. Overall, natural fibers achieved substantially higher retention rates (up to 80%) than synthetics, underscoring the importance of fabric-specific mordant selection to maximize dye–fiber interactions. These findings are supported by recent studies of bacterial carotenoid pigments as natural textile dyes, particularly for cotton. For instance, researchers applied yellow and orange carotenoid extracts to cotton fabrics, achieving satisfactory coloration and highlighting their potential as eco-friendly alternatives (Muneefa and Thirumalai, 2021). Similarly, Gurkok (2022) extracted an orange pigment from *Metabacillus idriensis* and successfully dyed cotton substrates for textile applications. Further experiments such as light fastness, rubbing fastness, color stability, and gray scaling will be useful to validate the industrial potential of the pigment.

## 4 Conclusion

We isolated a yellow-pigmented, psychrotrophic Gram-positive cocci, PS104, that could grow at 5–30 °C. It displayed 98.99% similarity to *Citricoccus parietis* strain 02-Je-010 indicating the PS104 as a *Citricoccus-*like species. HR-LCMS of the intracellular pigment extracted from PS104 confirmed a carotenoid-like pigment, possibly zeaxanthin, complemented by co-eluting metabolites that contributed to the observed yellow color and some unknown compound which needs further molecular level identification. The pigment yield was optimized using OFAT, RSM, and nanomaterials, resulting in a pigment-enhanced yield up to ∼3.9 times (∼290%) of basal media (26 mg/100 mL) to RSM media supplemented with 25 μg/mL of TiO_2_ (102 mg/100 mL). The pigmented extract appeared non-cytotoxic in preliminary study against MDCK cell lines, indicating good antioxidant activity and dyeing potential for natural fabrics. Beyond conventional optimization, our study contributed initial evidence of yellow pigment producing *Citricoccus*-like isolate PS104 from a new ecological niche in an Arctic fjord. This study also contributed to pigment enhancement strategy using nanomaterials and their preliminary applications in dyeing industry. However, further optimization of pigment production and textile dyeing may be required to achieve this at large-scale industrial production.

## Data Availability

The data presented in the study are deposited in the GenBank repository, accession number OQ257376.
