# Supplementary material for: Physico-chemical factors and nanomaterial-mediated optimization of pigment yield from a psychrotrophic marine Citricoccus-like isolate of an Arctic fjord
Source: Front Bioeng Biotechnol. 2026 Sep 25;14:1824301. doi: 10.3389/fbioe.2026.1824301 (PMC13651464; doi:10.3389/fbioe.2026.1824301)
Supplement: Supplementary file 1 [file Supplementaryfile1.pdf]

Physico-Chemical Factors and Nanomaterials Mediated Optimization of Pigment Yield from a Psychrotrophic Marine *Citricoccus*-like Isolate of Arctic Fjord

Rakshita Kaskar [1], Manisha Tiwari [1], Meghanath Prabhu [1], Savita Kerkar [1*] Dhermendra K. Tiwari[1, 2*]

[1]School of Biological Sciences and Biotechnology, Goa University, Taleigao Plateau, Goa, India – 403206,

[2]University Grant Commission (UGC), Bahadur Shah Zafar Marg, New Delhi – 110002

Supplementary material

Methods and statistical analysis

Identification and characterization of pigmented bacteria

We performed Gram staining of bacterial isolate using a Gram Staining Kit (K001, HiMedia, India) to confirm Gram characteristics (Coico, 1997). Cell morphology and size were examined by scanning electron microscopy (SEM). Briefly, 10 µL of freshly grown culture was spread on a 1 cm² slide, air-dried, and fixed overnight with 2.5% glutaraldehyde. Sample was washed with 0.1M sterile phosphate-buffered saline (pH 7.4), dehydrated sequentially with 30–90% acetone (10 min each), followed by 100% acetone for 30 min, and air-dried. The slide was sputter-coated with gold (SC7620 Mini Sputter Coater, Quorum Technologies, UK) and examined under a scanning electron microscope (Evo 18, Carl Zeiss, Germany) at 40,000X magnification (Bratbak, 2018).

Biochemical tests

Sulfur Indole Motility (SIM) test: The bacterial culture was stabbed into the SIM agar. The tubes were incubated at 30 ºC for 24-48 hours. The tubes were observed for hydrogen sulphide gas production and motility. The results were noted down before performing the indole test. 3 drops of Kovac's reagent were added to the surface of the agar. The surface was observed for the presence of a pink/red colour.

Methyl Red-Voges-Proskauer (MR-VP): The bacterial culture was inoculated into MR-VP broth and incubated at 30 ºC for 24-48 hours. MR-VP broth was further divided into two parts:

MR: 3-5 drops of methyl red were added to the medium. The tube was observed for colour change.

VP: 12 drops of Barritt's A and 2-3 drops of Barritt's B solution were added to the medium. The tube was shaken for 30 seconds and left undisturbed for 10-15mins. The tube was observed for colour change.

Citrate test: The bacterial culture was streaked on the slant containing Simmon's Citrate agar. The tubes were incubated at 30 ºC for 4-7 days. The surface was observed for colour change from green to blue.

Urease test: Tubes containing urease medium were prepared and inoculated with freshly grown culture. The tubes were incubated at 30 ºC for 48 hours to 7 days. The tubes were checked for any colour change over a period of 7 days.

Gelatinase test: The bacterial culture was stabbed into the gelatine agar and incubated at 30 ºC for 14 days. The tube was placed at 4 ºC to check for liquefaction on a daily basis.

Nitrate test: The bacterial culture was inoculated in nitrate broth and incubated at 30 ºC for 24 hours. Further, 6-8 drops of Reagent A (sulphanilic acid) and 6-8 drops of Reagent B (alpha-naphthylamine) were added and observed for any reaction. Zinc was added when no colour change was observed within a minute. The broth was observed for red precipitate formation for 3 minutes.

ONPG (o-nitrophenyl-β-D-galactopyranoside) test: The ONPG disc (HiMedia DD008) was placed into a sterile and dry test tube and 0.2 mL saline was added to it. An isolated test colony was inoculated into it and incubated at 30 ºC for 4 hours. The discs were observed for any colour development.

Oxidase test: The oxidase disc (HiMedia DD018) was inoculated with 10 µL of test culture. The disc was observed for colour change within 10 seconds of inoculation. Change beyond 10 seconds was considered oxidase negative.

Catalase test: A small amount of the isolated test colony was transferred onto a clean and dry glass slide using a nichrome loop. 3% H_2O_2 (2 drops) was added on the glass slide. The bacterial culture was observed for production of effervescence.

To further validate the identity of bacterium, we performed molecular identification by partial sequencing of the 16S rRNA gene. In brief, genomic DNA was isolated using the HiPurATM Bacterial Genomic DNA Purification Kit (HiMedia, India), and the 16S rRNA gene was amplified with universal primers; 27F and 1492R. The PCR product was sequenced by Sanger sequencing, analyzed using BLASTn against the NR database, and a phylogenetic tree was constructed in

MEGA X using the Maximum Likelihood method with 1000 bootstrap replicates. The sequence was deposited in GenBank.

Crude pigment extraction

The culture was grown in 100 mL nutrient broth at 25 ºC on a rotary shaker at 100 RPM for five days. Solvent extraction was carried out using polar solvents such as; ethanol, methanol, acetone, and non-polar solvents such as; hexane, chloroform, diethyl ether. For each solvent, 10 mL of five-day old culture was centrifuged at 10,000 RPM for 15 min at 4 ºC. The collected pellet was washed twice with distilled water and re-suspended in 5 mL solvents, vortexed and kept overnight at 4 ºC. On the subsequent day, the supernatant containing coloured pigment was collected by centrifugation at 10000 RPM. Absorption spectrum of this supernatant was recorded with UV-Visible spectrophotometer. The extracted pigment was stored at 4 ºC for subsequent experiments. (Chaturwedi et al., 2024). The stability of the pigment was evaluated with respect to pH, temperature and UV exposure. For pH stability testing, 1.0 mL aliquots of the pigment solution were adjusted from pH 2.0 to pH 14.0 using 1.0 M HCl or 1.0 N NaOH. Thermal stability was assessed by incubating 1 mL aliquots of the pigment solution at 20, 30, 40 and 50 ºC for 15 min in a water bath; samples were then returned to room temperature before measurement. UV stability was determined by exposing 4 mL pigment aliquots in a closed UV cabinet to shortwave UV (250 ± 10 nm) for 0, 5, 10, 15 and 30 minutes. After treatment, the absorption spectrum of each sample was recorded and compared to untreated controls to identify shifts in peak wavelength or reductions in absorbance indicative of pigment degradation.

OFAT (one-factor-at-a-time) screening

Screening of carbon and nitrogen sources

For detection of the suitable carbon source for pigment production, 100 mL of basal media was first supplemented by varying carbon sources viz.: fructose, glucose, glycerol, lactose, mannitol, mannose, sodium citrate, starch and sucrose, at 2% w/v concentration in 250 mL Erlenmeyer flasks, respectively (Elkenawy et al., 2017). All the cultured flasks were incubated on a rotary shaker at 100 RPM for three days at 25 ºC. The pigment was further extracted and the dry weight of the pigment was estimated. Subsequently, nitrogen sources viz. beef extract, casein, glycine, meat

extract, peptone, sodium nitrate, tryptone, urea and yeast extract at 2% w/v concentration, respectively, were added to the basal media containing suitable carbon source (Srimathi et al., 2017). The cultured flasks were incubated on a rotary shaker at 100 RPM for three days at 25 ºC. Pigment was further extracted and the dry weight of the pigment was estimated.

Screening for the optimal glycerol and yeast extract concentration

To study the effect of the carbon and nitrogen concentrations on the optimal production of the yellow pigment, the concentration of glycerol and yeast extract in the medium was altered respectively from 0.5 to 3% w/v (with a fixed increment of 0.5 units). The culture flasks were incubated on a rotary shaker at 100 RPM for three days at 25 ºC. The pigment was subsequently extracted and the dry weight of the pigment was determined.

Screening for the optimal salt concentration

In order to determine the optimum level of salt concentration for enhanced pigment production, the medium was supplemented with glycerol and yeast extract concentrations obtained from the above experiments and the salt concentration was varied from 0.5, 2, 4, 6, 8 and 10% w/v. The cultured flasks were incubated on a rotary shaker at 100 RPM for three days at 25 ºC. The pigment was extracted in methanol and the dry weight of the pigment was determined.

Screening of pH and temperature

The effect of pH on the production of pigment, was assessed by varying the pH of the basal media viz. pH 5, 5.5, 6, 6.5, 7, 7.5, 8 and 8.5, supplemented with the optimal carbon and nitrogen concentrations. The cultured flasks were incubated on a rotary shaker at 100 RPM for three days at 25 ºC. The pigment was further extracted and the dry weight of the pigment was recorded. Similarly, the incubation temperature was assessed from 5 to 30 ºC (with a fixed increment of 5 units) to understand the effect of temperature on the yield of the pigment. The culture was incubated on a rotary shaker at 100 RPM for three days at the respective temperatures. The pigment was further extracted in methanol and the dry weight was recorded.

Box-Behnken design for Response surface methodology (RSM)

Response surface methodology (RSM) was applied using Design Expert software, version 11.1.2.0 (Stat-Ease Inc., Minneapolis, MN, USA). Physical and chemical growth factors defining the pigment production; such as carbon source, nitrogen source, sodium chloride concentration and incubation temperature were considered for analysis. The Optimal conditions to improve pigment yield was performed using a randomized Box–Behnken design of RSM. Factor levels were coded as –1 (low) and +1 (high) (Venil et al., 2015). The design generated 27 experimental runs, comprising 24 axial points and 3 central points. Each run was conducted in 100 mL of culture medium under constant agitation (100 RPM) and incubated for three days. Analysis of variance (ANOVA) revealed significant effects of the experimental factors on pigment yield. Partitioning of variance into sum of square regression (SSR), sum of square error (SSE), and sum of square total (SST) confirmed the adequacy of the fitted model. Fisher's F-test indicated that the model was statistically significant, while the coefficient of determination (R^2) demonstrated a strong fit of the polynomial equation to the experimental data. The data generated through model-fitting was used for plotting the RSM, interference and contour plots. Contour plots derived from the significant factors illustrated their interactive influence on pigment production (Sharmila et al., 2019). Significant difference between the means $p < 0.05$ were considered.

Anti-oxidant activity

The DPPH (2,2-diphenyl-1-picrylhydrazyl) assay was performed to study the antioxidant activity of the pigments (Venil et al., 2016). Standard dilutions of ascorbic acid were made using 1 mg/mL of stock solution to compare the Radical Scavenging Activity (RSA) of the pigments to the test sample. Methanol was used as a control. The pigment was taken as a test sample. 2 mL of freshly prepared 0.05 mM DPPH solution was added to equal volume of standards, control and test, each. DPPH solution was prepared in methanol. The solutions were vortexed and kept in the dark for 30 minutes at room temperature. The absorbance at 517 nm was measured. Colour change from purple to yellow indicates ability to scavenge free radicals. This colour change was determined by measuring the absorbance at 517 nm. The percentage of radical scavenging activity was calculated by using the formula:

$$\text{RSA (\%)} = \frac{\text{Abs (C)} - \text{Abs(S)}}{\text{Abs(C)}} \times 100$$

RSA: Radical Scavenging Activity

Abs (C): Absorbance of control at 517 nm

Abs (S): Absorbance of test at 517 nm

Mordant-Assisted Dyeing of textile

To evaluate the potential of the pigment as a textile dye, seven commercially available fabrics i.e. cotton, jute, poplin, dacron, polyester, terry cotton, and viscose were selected and cut into uniform 2 × 2 cm pieces. The dyeing process involved three sequential steps; scouring, mordanting and dyeing. Scouring was performed by washing the fabric pieces in a 0.7% (w/v) solution of commercially available detergent (Henko, India) at 60 ^{o}C to remove dirt and impurities. This was followed by thorough rinsing with cold water and air drying the fabric overnight. The scoured fabrics were then placed in mordant solutions at 70 ^{o}C for 1 hour, followed by drying at room temperature overnight. Different types of mordants i.e. acidic (0.05% (w/v) acetic acid), alkali (0.05% (w/v) sodium bicarbonate), metal (0.05% (w/v) copper sulfate) and salt (0.05% (w/v) sodium chloride) were used to check the effectiveness. In order to dye the fabric, adding 10 mL (8mg/10 mL) of pigment dissolved in methanol was added to the pre-mordanted fabric at 60 ^{o}C until the fabric dried completely (Morales-Oyervides et al., 2017). Wash fastness of the dyed fabrics was assessed to determine dye retention with each mordant. Dyed samples were washed in 0.7% (w/v) soap solution at room temperature and the absorbance of the wash solution was measured in a UV-Visible spectrophotometer, at the pigment's maximum absorption wavelength ($\lambda_{\max}$), to quantify pigment loss. Appropriate blank (soap solution with (8mg) of the pigment) was used for the experiment (Krishna et al., 2008).

$$\text{Dye retention capacity (\%)} = (\text{Abs}_{(initial)} - \text{Abs}_{(final)}) \times 100$$

Abs $_{(initial)}$: Absorbance of soap solution containing pigment

Abs $_{(final)}$: Absorbance of washed fabric solution

Supplementary results

Experimental design and statistical analysis for RSM

Box Behnken Design (BBD) was employed to assess the pigment yield and interaction among the physico-chemical factors such as glycerol concentration (Factor A), yeast extract (Factor B), NaCl (Factor C) and incubation temperature (Factor D). Parameters with coded high and low values were set for each factor.

For statistical analysis, a total of 27 runs with 24 axial points and 3 replications of central point were selected. Supplementary Table S4 displays experimental and predicted values of the response obtained for all 27 runs. As seen in Supplementary Table S4 response varied from 14 mg to 74 mg, which indicates ratio of maximum: minimum as 5.285. Since the ratio was below 10, no transformation was needed.

Different statistical tests such as lack of fit test, the sequential model (sum of squares) and model summary statistics were applied to test the efficacy of the model to demonstrate maximum yield of the pigment. Supplementary Table S5 indicates the results for these analysed tested parameters and the maximum predicted experimental yield of the pigment. As seen in the Supplementary Table S5, with reference to the F and p values obtained, cubic model was aliased and quadratic model was proposed.

The regression analysis was carried out to fit the mathematical models to the experimental data, aiming at an optimal region for the responses studied. The significance of each coefficient is listed in Supplementary Table S6. Analysis of Variance (ANOVA) was applied to determine the significance value of the model indicates that the model is significant with F-value of 26.18 and p-value of <0.0001. The model F-value of 26.18 implies that the model is significant. There is 0.01% chance that an F-value this large could occur due to noise. It is also understood, that p-value of less than 0.05 indicate model terms are significant. From Supplementary Table S6, it could be determined that three linear coefficients (A, C, and D), three interactive coefficients (AB, AC, and AD) and four quadratic coefficients (A^2, B^2, C^2 and D^2) were significant with p-value > 0.05 whereas one linear coefficient (B) and two interactive coefficients (BC, BD and CD) were insignificant. Likewise, lack-of-fit F-value was found to be 17.07 which also indicates high ability

of the model to predict the results. A second order polynomial quadratic equation was defined in coded factors as represented by Equation 1. The equation in terms of coded factors can be used to make predictions about the response for given levels of each factor. It is useful in identifying the relative impact of the factors by comparing the factor coefficients.

$$Dry\ weight = 73.33 + 3.17A + 0.5B - 9.67C - 8.0D - 14.5AB - 7.5AC - 10.5AD + 2.0BC - 1.0BD - 1.5CD - 13.75A^2 - 12.25B^2 - 19.0C^2 - 22.0D^2 \quad \text{.........Eq.1}$$

In order to determine fitness of the model, the coefficient of determination (R^2) was calculated and found to be 0.9683. The R^2 value closer to 1.00 indicates the model is stronger and better to predict the response. The adjusted determination coefficient (adjusted R^2) of 0.9313 and predicted determination coefficient (predicted R^2) of 0.8187 gives the difference of less than 0.2 confirming that and predicted R^2 is in reasonable agreement with adjusted R^2. Adequate precision is measured by signal to noise ratio. It compares the range of the predicted values at the design points to the average prediction error. A ratio greater than four is desirable. Ratio of 18.425 was obtained for the present model, which indicates an adequate signal confirming model's ability to navigate the design space.

The geometry of the contour plot shows the combined influence of the independent variables on the response parameter. All reaction surface plots and the resulting contour map have a design stage. As seen in the Figure 3G, contour plots show highest response obtained is located within the design boundaries. The structure of the contour plot predicts whether the interaction between variables is relevant or not. Elliptical contours are acquired when absolute shared interactions exist between operational variables. If the contour is elliptical then mutual combination of the variables is more prominent in determining the response whereas circular contours indicate the variables when present in combination might be less prominent in affecting the response. A three-dimensional response surface curve determines the interaction effects and ideal value of the variables which will lead to higher response. Supplementary Figure S4 displays response surface graphs obtained during the study. The response i.e. dry weight of the pigment increases with increase in carbon concentration, nitrogen concentration, NaCl concentration and temperature till a particular point then further shows a steady decline.

The highest pigment yield of 76.752 mg was suggested by the model with the factors, 2.316% carbon concentration, 1.419% nitrogen concentration, 3.603% NaCl concentration and incubation temperature of 14.104ºC. A second verification experiment was performed in order to compare and confirm the ideal medium conditions suggested by the model to obtain maximum yield. The model was successful in determining the factors as the response obtained through the new medium composition increased 1.5 times, producing 78 mg/100 mL of the pigment (Figure 4A). The Percent Error was found to be 1.69% by the Equation 2. Overall, the yield of the pigment increased threefold by Response Surface Methodology.

$$\text{Percent Error} = \frac{\text{Experimental yield} - \text{Predicted yield}}{\text{Predicted yield}} \times 100; \quad \ldots\ldots \text{Eq. 2}$$

Supplementary figures

(a)

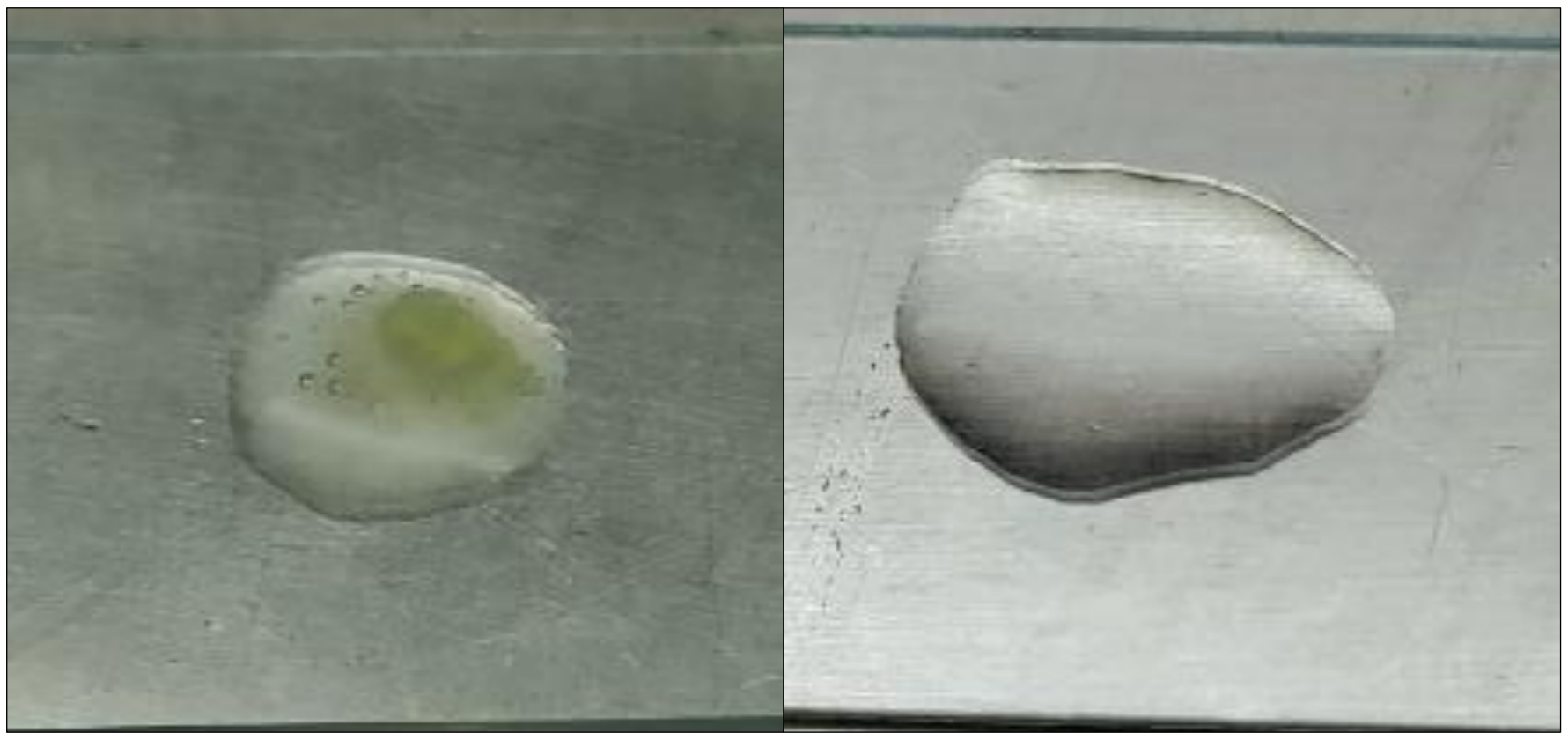

(b)

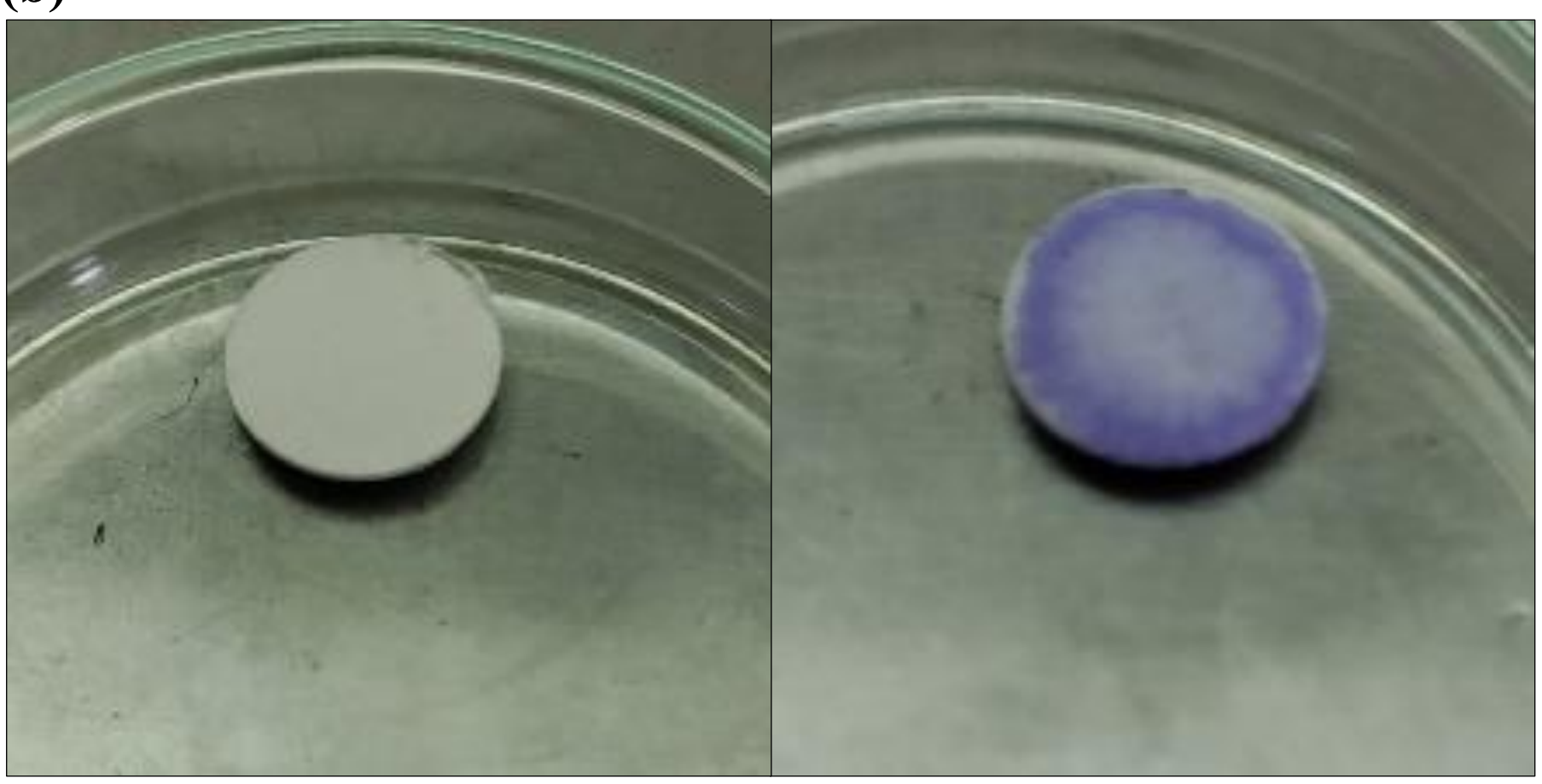

(c)

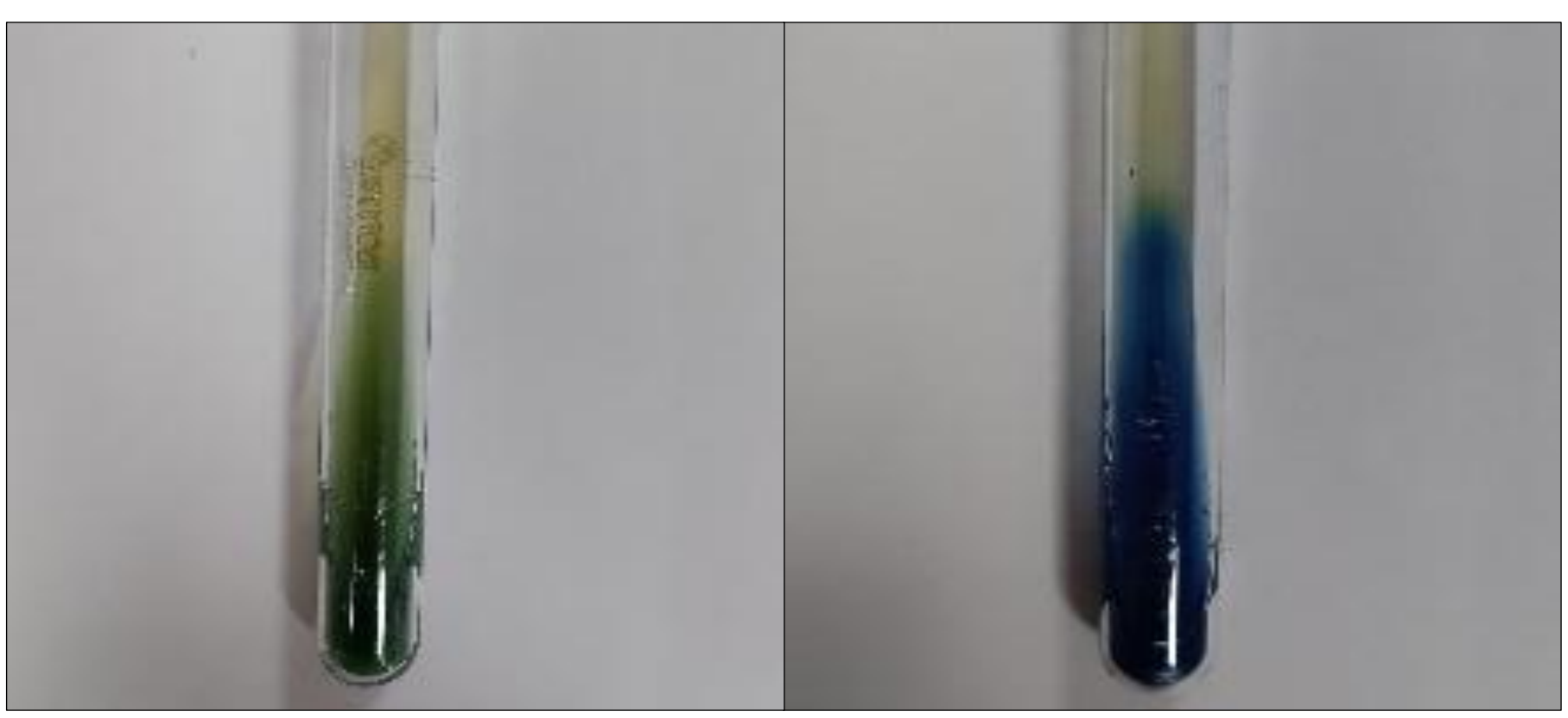

Figure S1: Test and control for, **a)** catalase. **b)** oxidase. **c)** citrate tests respectively

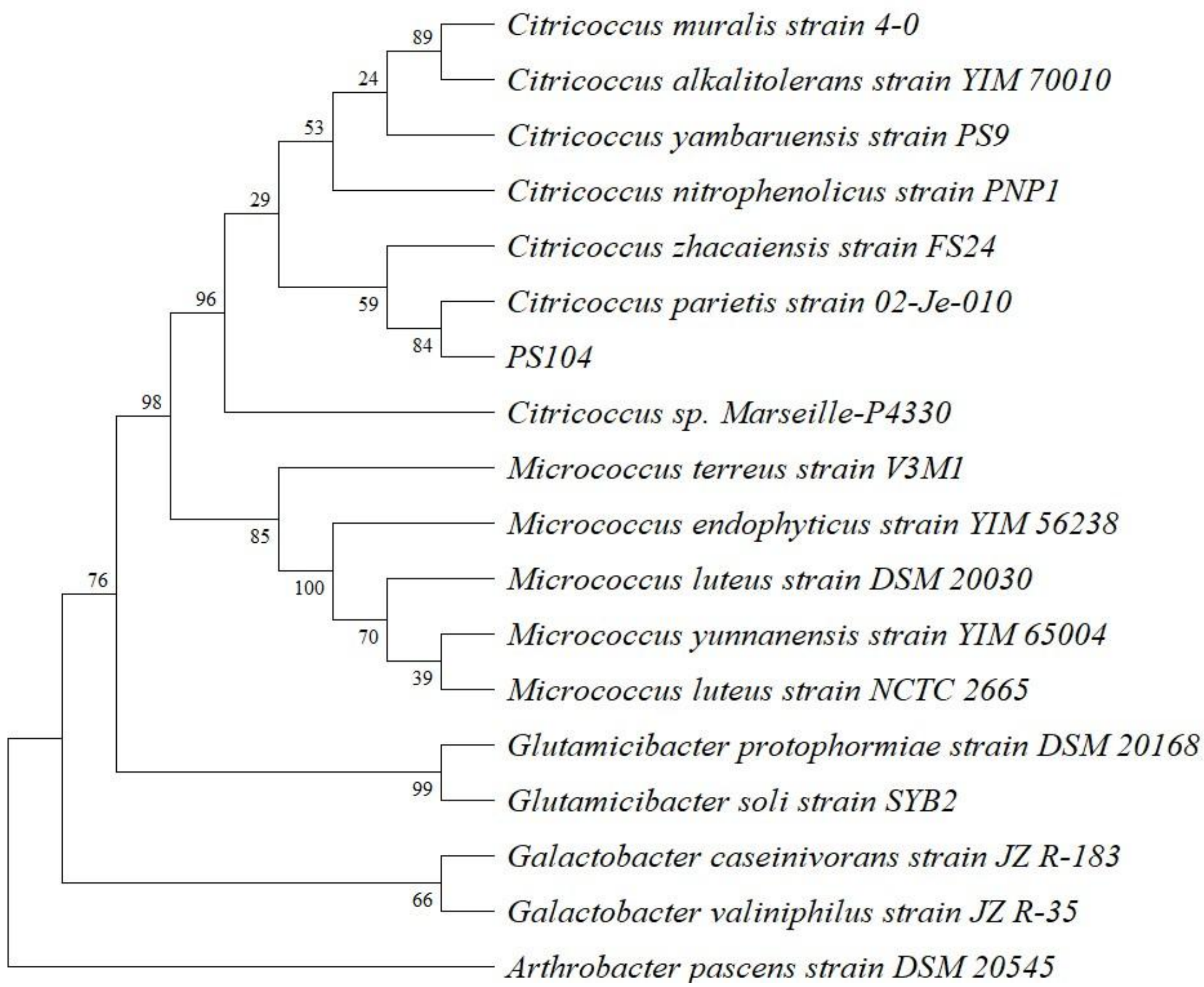

Figure S2: Phylogenetic tree of PS104 constructed by Maximum-Likelihood method in MEGA.

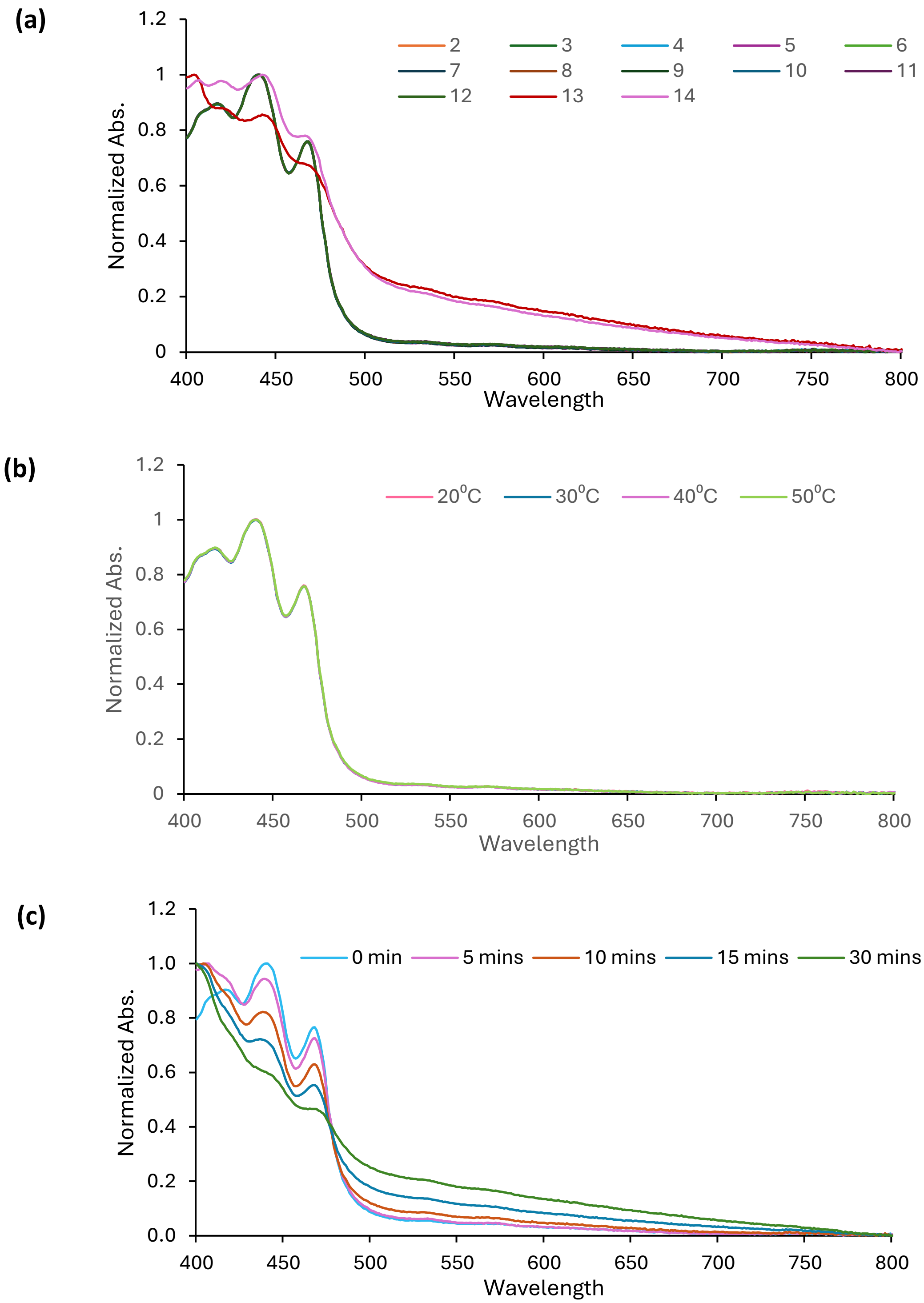

Figure S3: Pigment stability at different parameters **(a)** pH 2.0 to pH 14, **(b)** temperature 20, 30, 40 and 50 °C and **(c)** exposure to UV for 0, 5, 10, 15 and 30 mins.

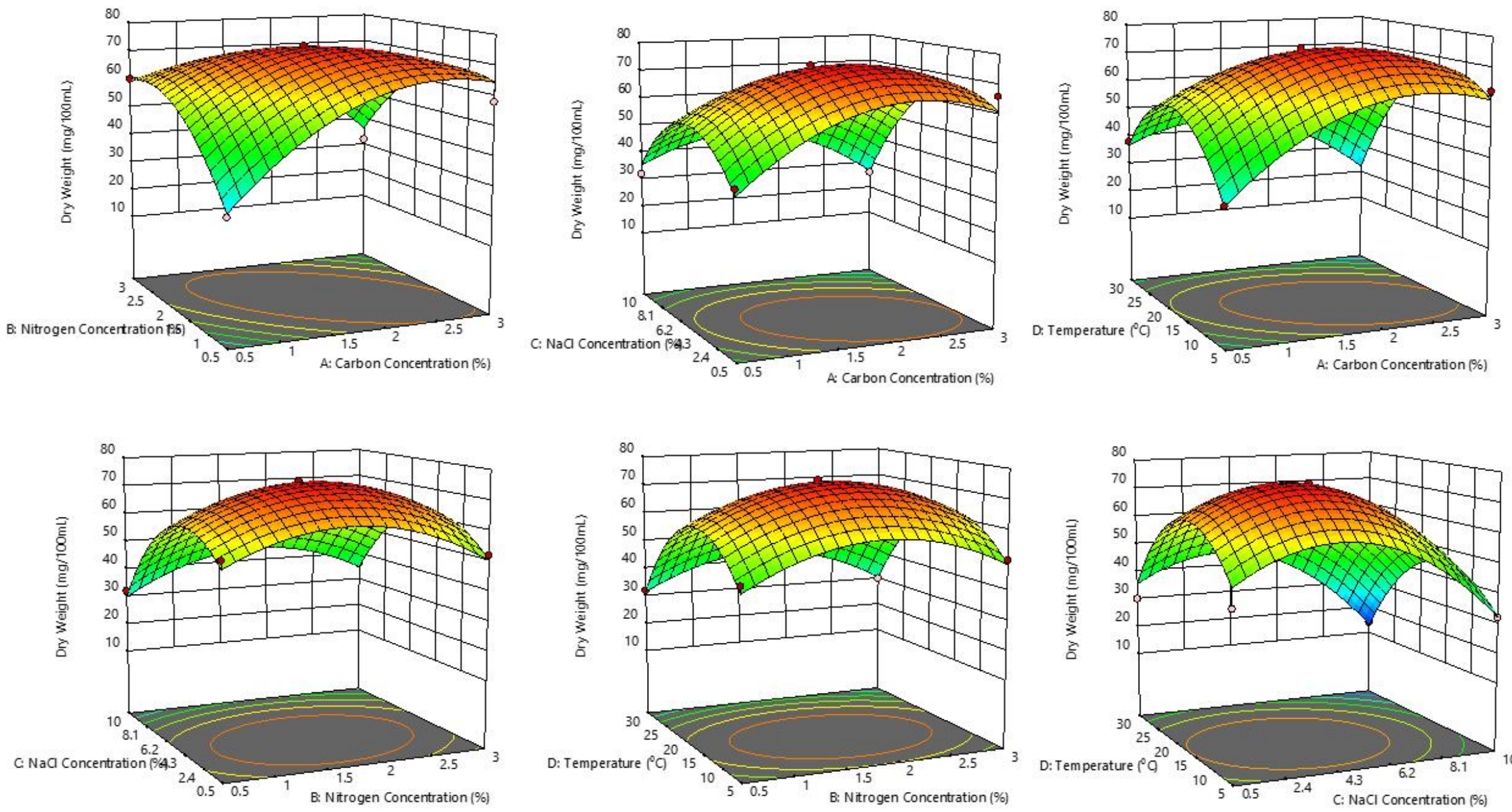

Figure S4: Three-dimensional surface plots indicating highest response with combinations of significant variables. Figure 4 in main text represent corresponding data.

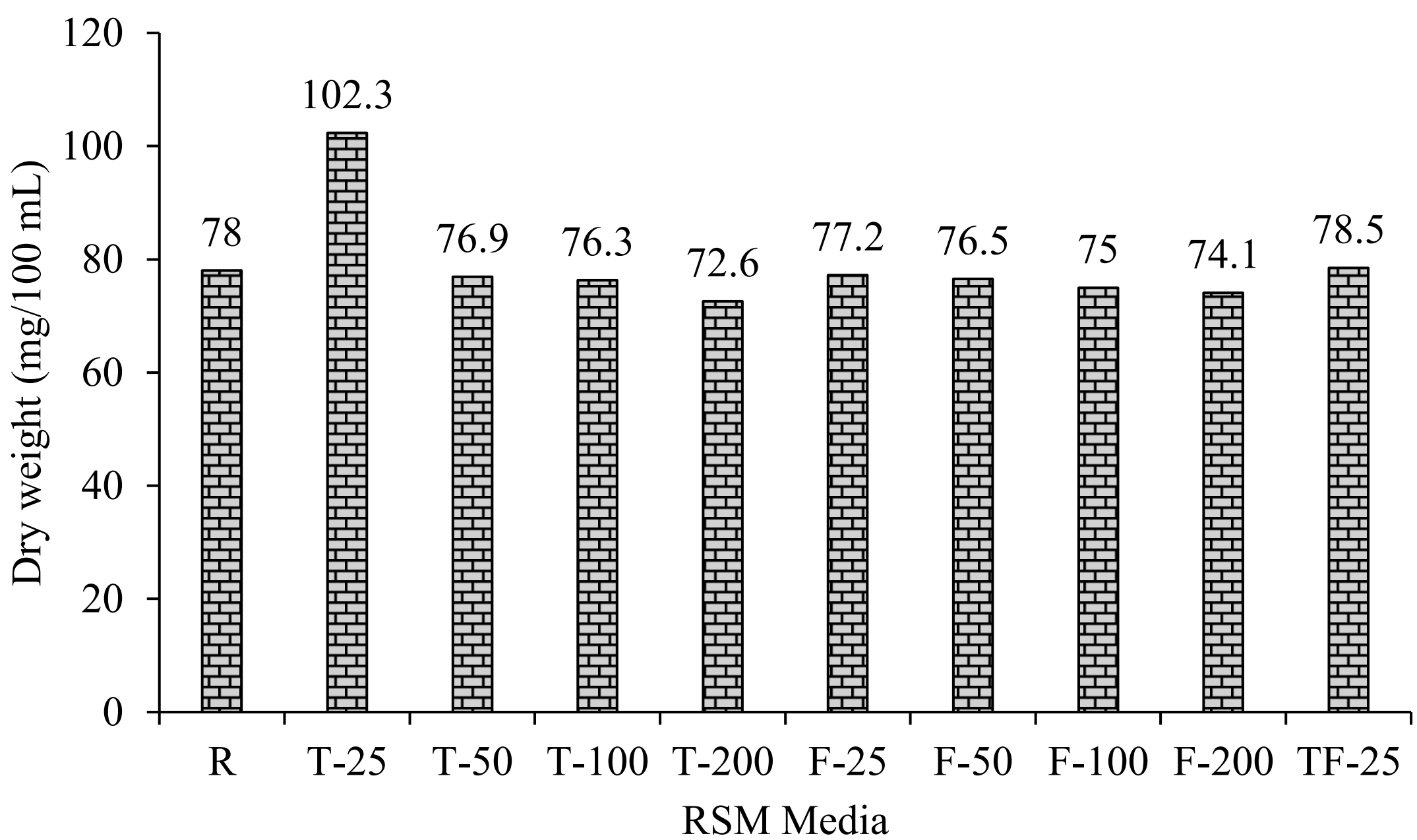

Figure S5: Pigment Yield from RSM Medium with photoactive nanomaterials (T: TiO_2; F: Fe_3O_4) at varying concentrations (μg/mL)

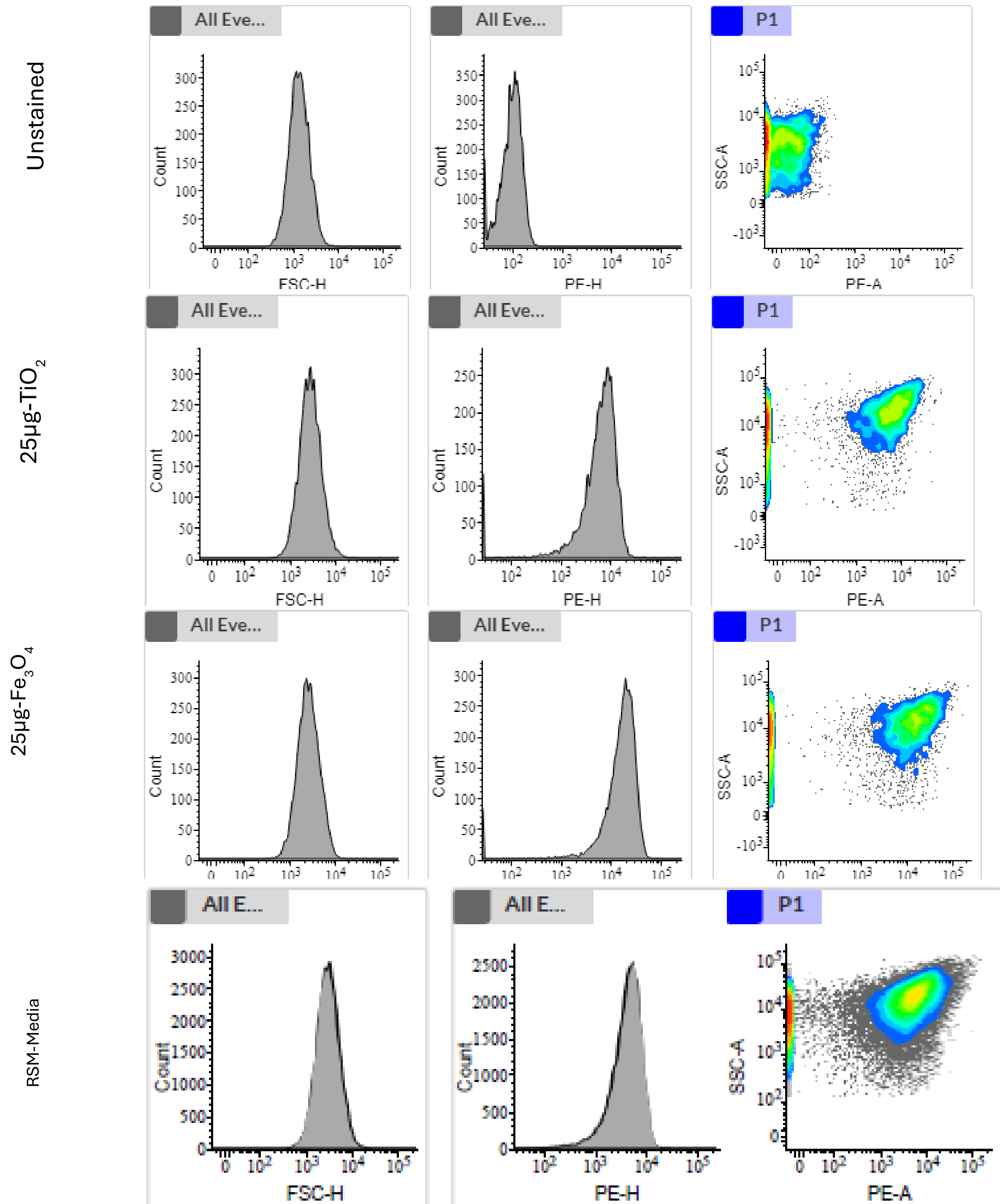

Figure S6: Flow cytometry plots of bacterial cell stained with PI. indicating cytotoxic effect of 25 µg/mL of TiO_2 and 25 µg/mL of Fe_3O_4 on the cell viability. The graph indicates there is no prominent effect on the viability of bacterial cells due to the nanomaterial.

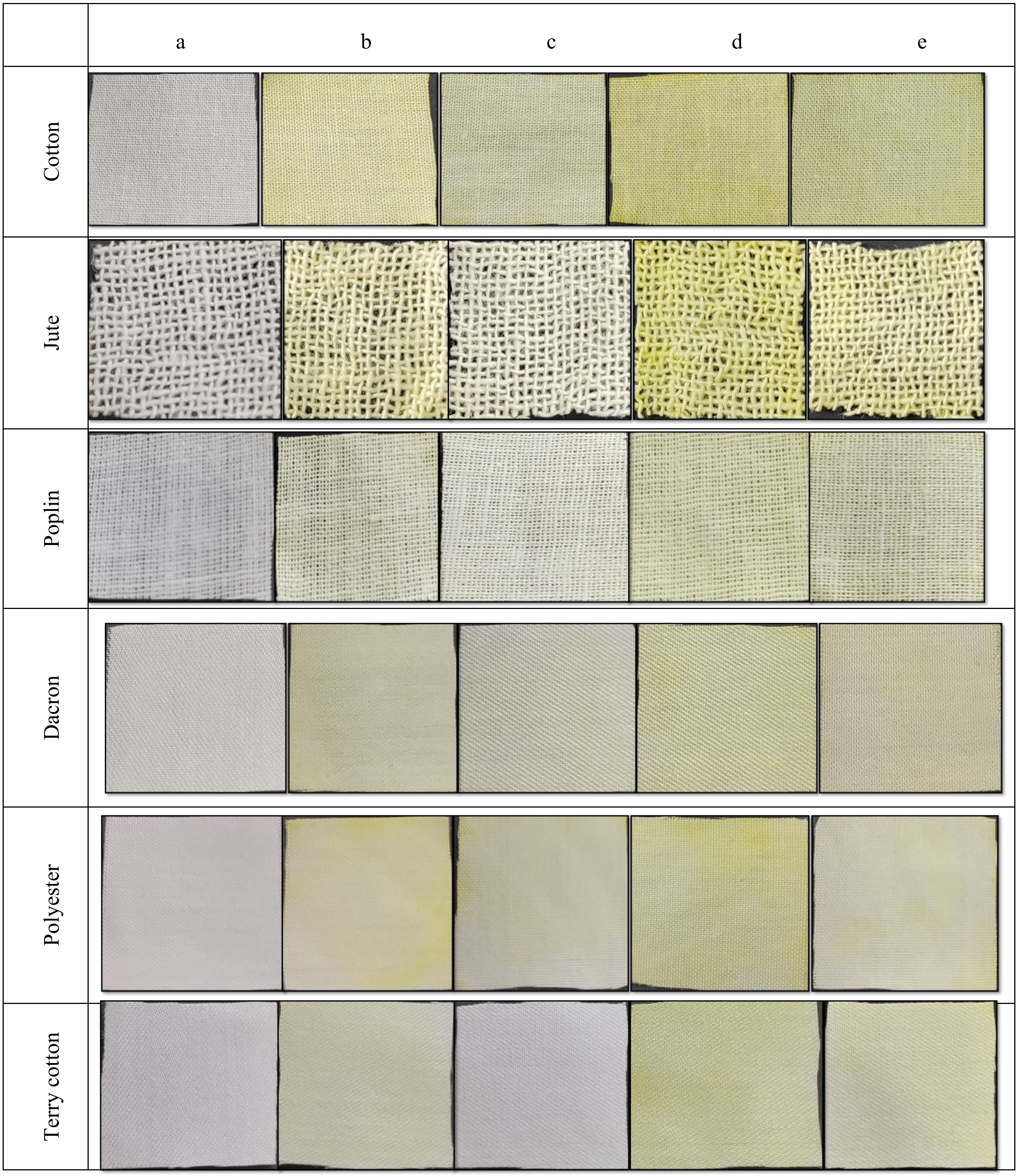
a
b
c
d
e
Cotton
Jute
Poplin
Dacron
Polyester
Terry cotton

Viscose	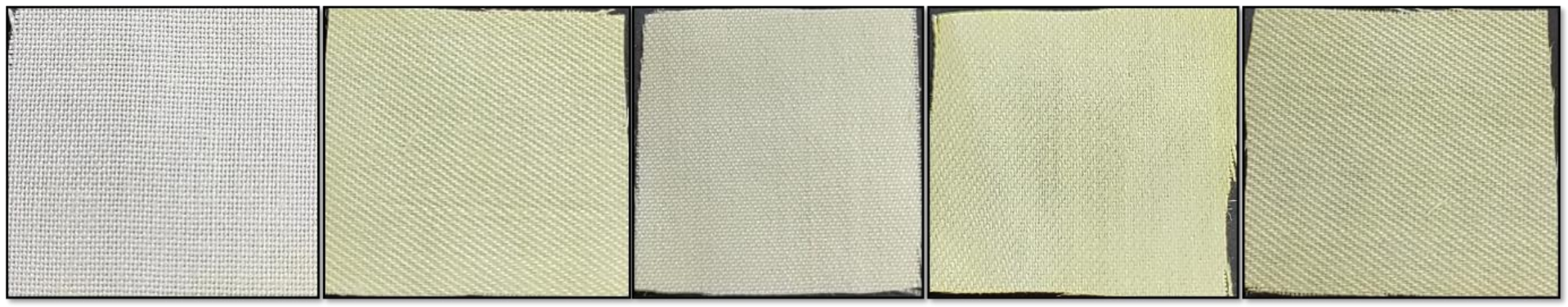

Figure S7: Fabrics dyed using the best mordants. (a) control fabric; (b) fabric dyed with the pigment; (c) post-wash fabric dyed with the pigment; (d) pre-mordanted fabric dyed with the pigment; (e) post-wash pre-mordanted fabric dyed with the pigment. We used copper sulphate, sodium bicarbonate, acetic acid and sodium chloride as mordant. The pre-mordanted fabrics shown in the above image are using the best mordant (cotton, poplin and terry cotton with alkali; jute with metal and dacron, polyester and viscose with acid). Images with other mordant are not shown here.

Supplementary Tables

Table S1: Media composition for 1000mL.

Ingredients	Nutrient Agar	Basal Media	OFAT Media	RSM Media	25% ZMA
Peptone	5	–	–	–	1.25
Sodium chloride	5	5	60	36.03	4.86
Beef extract	1.5	–	–	–	–
Yeast extract	1.5	20	15	14.19	0.25
Glucose	–	20	–	–	–
Glycerol	–	–	20	23.16	–
Disodium hydrogen phosphate	–	6	6	6	0.002
Potassium dihydrogen phosphate	–	3	3	3	–
Agar	15	–	–	–	15
Ferric citrate	–	–	–	–	0.025
Magnesium chloride	–	–	–	–	1.475
Sodium sulfate	–	–	–	–	0.81
Calcium chloride	–	–	–	–	0.45
Potassium chloride	–	–	–	–	0.1375
Sodium bicarbonate	–	–	–	–	0.04
Potassium bromide	–	–	–	–	0.02
Strontium chloride	–	–	–	–	0.0085
Boric acid	–	–	–	–	0.0055
Sodium silicate	–	–	–	–	0.001
Sodium fluoride	–	–	–	–	0.0006
Ammonium nitrate	–	–	–	–	0.0004
Disodium phosphate	–	–	–	–	0.002

Table S2: Biochemical test results of PS104

Tests	Results
Sulphur reduction test	Negative
Indole production test	Negative
Motility test	Negative
Methyl red test	Negative
Voges-Proskauer test	Positive
Citrate test	Positive
Urease test	Negative
Gelatinase test	Negative
Nitrate test	Negative
ONPG test	Negative
Oxidase test	Positive
Catalase test	Positive

Table S3: List of compounds producing yellow colour and compounds un-identified by HR-LCMS analysis

Retention time	Mass	Compound Name	Compound formula
1.629	100.064	N-Nitrosopyrrolidine	$C_4H_8N_2O$
1.922	100.064	N-Nitrosopyrrolidine	$C_4H_8N_2O$
3.64	111.0685	Trimethyloxazole	C_6H_9NO
4.537	161.1207	5,6,7,8-Tetrahydro-2,4 dimethylquinoline	$C_{11}H_{15}N$
8.236	183.1104	2,4-Diethyl-5-propylthiazole	$C_{10}H_{17}NS$
10.872	335.1107	Benfluralin	$C_{13}H_{16}F_3N_3O_4$
20.274	197.12	2-(3-Phenylpropyl)pyridine	$C_{14}H_{15}N$
21.431	98.0374	5-Hydroxy-4-pentenoic acid d lactone	$C_5H_6O_2$
22.192	568.431	(3S,3'S,all-E)-Zeaxanthin	$C_{40}H_{56}O_2$
25.31	281.2685	Oleamide	$C_{18}H_{35}NO$
25.597	314.242	9,10-DiHOME	$C_{18}H_{34}O_4$
3.308	-	Unidentified	-
3.801	-	Unidentified	-
6.587	-	Unidentified	-
18.058	-	Unidentified	-
18.673	-	Unidentified	-
19.308	-	Unidentified	-
19.358	-	Unidentified	-
19.621	-	Unidentified	-
20.24	-	Unidentified	-
20.274	-	Unidentified	-

23.334	-	Unidentified	-
23.635	-	Unidentified	-
24.775	-	Unidentified	-
27.348	-	Unidentified	-

Table S4: Effect of Carbon Source on Pigment Production by PS104 (One-way ANOVA with Tukey's HSD Post-hoc Analysis; Group Comparisons vs Starch). ΔM values are calculated relative to the highest-yielding control (glycerol for carbon sources).

Carbon Source	Mean Pigment Yield (mg/100 mL)	ΔM vs Glycerol	95% CI (ΔM)	Tukey's q	p value
Glycerol	33.67 ± 0.47	-	–	–	–
Glucose	25.33 ± 0.47	−8.33	[−10.07, −6.59]	23.72	<0.0001
Fructose	19.67 ± 0.47	−14.00	[−15.74, −12.26]	39.84	<0.0001
Mannose	17.33 ± 0.47	−16.33	[−18.07, −14.59]	46.49	<0.0001
Sucrose	13.00 ± 0.82	−20.67	[−22.41, −18.93]	58.82	<0.0001
Lactose	11.67 ± 0.47	−22.00	[−23.74, −20.26]	62.61	<0.0001
Sodium citrate	10.33 ± 0.47	−23.33	[−25.07, −21.59]	66.41	<0.0001
Mannitol	7.33 ± 0.47	−26.33	[−28.07, −24.59]	74.95	<0.0001
Starch	0.00 ± 0.00	−33.67	[−35.41, −31.93]	95.82	<0.0001

Table S5: Effect of Nitrogen Source on Pigment Production by PS104 (One-way ANOVA with Tukey's HSD Post-hoc Analysis; Group Comparisons vs Urea). ΔM values are calculated relative to the highest-yielding control (yeast extract for nitrogen sources).

Nitrogen Source	Mean Pigment Yield (mg/100 mL)	ΔM vs Yeast Extract	95% CI (ΔM)	Tukey's q	p value
Yeast Extract	38.00 ± 0.00	-	–	–	–
Peptone	31.00 ± 0.82	−7.00	[−8.56, −5.44]	22.28	<0.0001
Beef Extract	27.67 ± 0.47	−10.33	[−11.89, −8.78]	32.88	<0.0001
Casein	22.67 ± 0.47	−15.33	[−16.89, −13.78]	48.79	<0.0001
Meat Extract	21.33 ± 0.47	−16.67	[−18.22, −15.11]	53.03	<0.0001
Glycine	14.67 ± 0.47	−23.33	[−24.89, −21.78]	74.24	<0.0001
Sodium Nitrate	11.00 ± 0.00	−27.00	[−28.56, −25.44]	85.91	<0.0001
Tryptone	9.33 ± 0.47	−28.67	[−30.22, −27.11]	91.22	<0.0001
Urea	0.00 ± 0.00	−38.00	[−39.56, −36.44]	120.93	<0.0001

Table S6: The Box–Behnken response surface design for pigment production and corresponding response values

Run	A: Carbon concentration	B: Nitrogen concentration	C: NaCl concentration	D: Temperature	Response: Dry weight		Residual
					Actual	Predicted	
1	0	0	0	0	74	73.33	0.667
2	0	0	1	-1	32	32.17	-0.1667
3	0	0	-1	-1	42	48.5	-6.5
4	-1	0	0	-1	32	31.92	0.0833
5	0	1	-1	0	52	50.25	1.75
6	-1	1	0	0	60	59.17	0.8333
7	0	-1	-1	0	56	53.25	2.75
8	0	1	0	-1	50	48.58	1.42
9	1	1	0	0	32	36.5	-4.5
10	1	0	0	-1	62	59.25	2.75
11	0	-1	0	-1	48	45.58	2.42
12	0	1	0	1	30	30.58	-0.5833
13	1	0	0	1	26	22.25	3.75
14	0	0	0	0	72	73.33	-1.33
15	0	0	-1	1	30	35.5	-5.5
16	1	0	-1	0	66	60.92	5.08
17	-1	0	1	0	32	35.25	-3.25
18	-1	-1	0	0	28	29.17	-1.17
19	-1	0	0	1	38	36.92	1.08

20	1	0	1	0	26	26.58	-0.5833
21	0	0	0	0	74	73.33	0.6667
22	1	-1	0	0	58	64.5	-6.5
23	0	0	1	1	14	13.17	0.8333
24	0	1	1	0	36	34.92	1.08
25	0	-1	0	1	32	31.5	0.4167
26	-1	0	-1	0	42	39.58	2.42
27	0	-1	1	0	32	29.92	2.08

Table S7: Sequential model fitting for yield of the pigment

**Sequential Model Sum of Squares**					
Source	Sum of Squares	dF	Mean Square	F-value	p-value
Mean vs Total	51221.33	1	51221.33		
Linear vs Mean	2012.67	4	503.17	2.11	0.1142
2FI vs Linear	1536	6	256	1.1	0.4035
Quadratic vs 2FI	3487.67	4	871.92	45.43	<0.0001
Cubic vs Quadratic	127.33	8	15.92	0.6181	0.7395
Residual	103	4	25.75		
Total	58488	27	2166.22		
**Lack of Fit Tests**					
Source	Sum of Squares	dF	Mean Square	F-value	p-value
Linear	5251.33	20	262.57	196.93	0.0051
2FI	3715.33	14	265.38	199.04	0.005
Quadratic	227.67	10	22.77	17.07	0.0566
Cubic	100.33	2	50.17	37.62	0.0259
Pure Error	2.67	2	1.33		
**Model Summary Statistics**					
Source	Std. Dev	R^2	Adjusted R^2	Predicted R^2	PRESS

Linear	15.45	0.277	0.1455	0.0425	6958.06
2FI	15.24	0.4883	0.1686	0.1183	6407.26
Quadratic	4.38	0.9683	0.9313	0.8187	1317.36
Cubic	5.07	0.9858	0.9079	-0.9891	14454

Table S8: Analysis of the variance and regression coefficients of the calculated surface quadratic model

**Source**	**Sum of Squares**	**dF**	**Mean Square**	**F-value**	**p-value**	
Model	7036.33	14	502.6	26.18	<0.0001	**Significant**
A-Carbon concentration	120.33	1	120.33	6.27	0.0277	
B-Nitrogen concentration	3	1	3	0.1563	0.6995	
C-NaCl concentration	1121.33	1	1121.33	58.42	<0.0001	
D-Temperature	768	1	768	40.01	<0.0001	
AB	841	1	841	43.81	<0.0001	
AC	225	1	225	11.72	0.005	
AD	441	1	441	22.98	0.0004	
BC	16	1	16	0.8336	0.3792	
BD	4	1	4	0.2084	0.6562	
CD	9	1	9	0.4689	0.5065	
A^2	1008.33	1	1008.33	52.53	<0.0001	
B^2	800.33	1	800.33	41.7	<0.0001	
C^2	1925.33	1	1925.33	100.31	<0.0001	
D^2	2581.33	1	2581.33	134.48	<0.0001	
Residual	230.33	12	19.19			
Lack of Fit	227.67	10	22.77	17.07	0.0566	**not significant**
Pure Error	2.67	2	1.33			

Cor Total	7266.67	26				

Table S9: Pigment yield with RSM media supplemented with varying concentrations of photoactive nanomaterials.

RSM Media	Dry weight (mg/100ml)
RSM	78.0
**RSM+TiO_2-25 µg/mL**	**102.3**
RSM+ TiO_2-50 µg/mL	76.9
RSM+ TiO_2-100 µg/mL	76.3
RSM+ TiO_2-200 µg/mL	72.6
RSM+Fe_3O_4-25 µg/mL	77.2
RSM+ Fe_3O_4-50 µg/mL	76.5
RSM + Fe_3O_4-100 µg/mL	75.0
RSM+ Fe_3O_4-200 µg/mL	74.1
RSM + TiO_2- 25 Fe_3O_4- 25 µg/mL	78.5

Table S10: Radical scavenging activity of the standard (ascorbic acid), test and control (Methanol).

Concentration (µg/mL)	Absorbance at 517nm	Radical Scavenging Activity (%)
1000	0.019	96.20
100	0.021	95.80
10	0.054	89.22
9	0.098	80.43
8	0.142	71.65
7	0.186	62.87
6	0.231	53.89
5	0.266	46.90
4	0.310	38.12
3	0.352	29.74
2	0.390	22.45
1	0.445	11.11
Pigment sample (9.5)	0.069	86.22
Control (Methanol)	0.501	0
